# Head‐to‐tail cyclization of side chain‐protected linear peptides to recapitulate genetically‐encoded cyclized peptides

**DOI:** 10.1002/pep2.24254

**Published:** 2022-01-06

**Authors:** Samir Bouayad‐Gervais, Daniel J. St‐Cyr, Mathieu Courcelles, Éric Bonneil, Florence H. Gohard, Pierre Thibault, William C. Earnshaw, Mike Tyers

**Affiliations:** ^1^ Department of Medicine, Institute for Research in Immunology and Cancer Université de Montréal Montréal Canada; ^2^ Wellcome Trust Centre for Cell Biology, Institute of Cell Biology University of Edinburgh Edinburgh UK; ^3^ Present address: X‐Chem Inc., 7171 Frederick‐Banting Montréal, Québec H4S 1Z9 Canada

## Abstract

Genetically‐encoded cyclic peptide libraries allow rapid *in vivo* screens for inhibitors of any target protein of interest. In particular, the Split Intein Circular Ligation of Protein and Peptides (SICLOPPS) system exploits spontaneous protein splicing of inteins to produce intracellular cyclic peptides. A previous SICLOPPS screen against Aurora B kinase, which plays a critical role during chromosome segregation, identified several candidate inhibitors that we sought to recapitulate by chemical synthesis. We describe the syntheses of cyclic peptide hits and analogs via solution‐phase macrocyclization of side chain‐protected linear peptides obtained from standard solid‐phase peptide synthesis. Cyclic peptide targets, including cyclo‐[CTWAR], were designed to match both the variable portions and conserved cysteine residue of their genetically‐encoded counterparts. Synthetic products were characterized by tandem high‐resolution mass spectrometry to analyze a combination of exact mass, isotopic pattern, and collisional dissociation‐induced fragmentation pattern. The latter analyses facilitated the distinction between targets and oligomeric side products, and served to confirm peptidic sequences in a manner that can be readily extended to analyses of complex biological samples. This alternative chemical synthesis approach for cyclic peptides allows cost‐effective validation and facile chemical elaboration of hit candidates from SICLOPPS screens.

## Graphical abstract

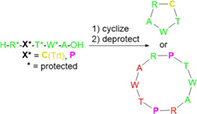

## 1 INTRODUCTION

Access to complex chemical matter is critical for the discovery of bioactive compounds that serve as chemical probes in biomedical research and as entry points for drug discovery.^[^ ^1^ ^]^ Traditionally, either natural product extracts or synthetic chemistry have been used to generate compound libraries for high‐throughput screens based on either *in vitro* biochemical assays or *in vivo* phenotypic readouts. New technologies promise to accelerate interrogation of bioactive chemical space including diversity‐oriented synthesis,^[^ ^2^ ^]^ DNA‐encoded libraries,^[^ ^3^ ^]^ combinatorial enzymatic biosynthesis of natural product‐like compounds,^[^ ^4^, ^5^ ^]^ and artificial intelligence‐based virtual screening.^[^ ^6^ ^]^ Genetically‐encoded peptide libraries, as produced in bacteriophage, bacteria, yeast, and *in vitro* formats, allow access to tremendous combinatorial diversity and have been successfully deployed against many important drug targets.^[^ ^7^ ^]^ While linear peptides represent the simplest case for both genetic encoding and chemical synthesis, linear peptides suffer from several liabilities including entropic penalties during binding due to backbone flexibility, poor cellular uptake, rapid proteolytic degradation, and notoriously difficult conversion to more drug‐like compounds.^[^ ^8^ ^]^ Head‐to‐tail backbone (homodetic) cyclization represents a simple yet powerful peptide modification that imparts rigidified structure, biorelevant turn conformations, increased proteolytic stability, and improved membrane permeability.^[^ ^9^ ^]^ Furthermore, among scaffolds capable of targeting protein–protein interactions (PPIs), cyclic peptides offer both biogenic and chemical synthetic accessibility.^[^ ^10^, ^11^ ^]^

Several strategies have been developed for the genetically‐encoded biosynthesis of cyclic peptides.^[^ ^9^ ^]^ An approach called flexible *in vitro* translation uses re‐engineered enzymes to produce modified tRNA species that can be used to program *in vitro* translation of an mRNA template for introduction of a backbone ester bond to promote macrocyclization.^[^ ^12^ ^]^ Another strategy entails the reprogramming of ribosomally synthesized and post‐translationally modified peptides, a class of natural product based on genetically‐encoded linear scaffolds that can be cyclized and extensively modified *in vivo*.^[^ ^13^ ^]^ A general approach called Split Intein Circular Ligation of Protein and Peptides (SICLOPPS) exploits autocatalytic protein splicing of genetically‐encoded linear precursor polypeptides to generate circular excised peptides; sequence length and composition can vary across a broad range, limited by the need for at least one Thr, Ser, or Cys residue to enable cyclization as well as the steric and electronic nature of the amino acid in the −1 position.^[^ ^14^, ^15^, ^16^ ^]^ These various genetic routes to cyclic peptides offer compelling advantages that include massive sequence diversity, ease of library construction, and low‐cost genetic screens. Importantly, candidate hits from genetically‐encoded cyclic peptide screens must be validated for bioactivity with chemically‐synthesized macrocycles.^[^ ^16^, ^17^ ^]^

We previously reported the use of SICLOPPS to generate cyclic peptides that modulate the interaction of the Aurora B kinase and the Inner Centromere Protein, which form part of the Chromosomal Passenger Complex (CPC), a key mitotic regulator.^[^ ^18^, ^19^ ^]^ Given its key role in cell division, the CPC represents a potential anti‐cancer target.^[^ ^20^ ^]^ The synthesis of genetically‐encoded hits from this SICLOPPS screen required simultaneous incorporation of multiple residues with innate chemical reactivity (i.e., Cys, Trp, Glu, Lys), precluding use of simple linear precursors obtained from canonical Wang supports during fluorenylmethoxycarbonyl/tert‐butyl (Fmoc/t‐Bu) solid‐phase peptide synthesis (SPPS). To circumvent this issue, we employed a 2‐chlorotrityl (2‐ClTrt) resin in conjunction with mild peptide release conditions to preserve side‐chain protection.^[^ ^21^ ^]^ Such linear peptides are suitable for active ester formation at high‐dilution to generate the macrocycle.^[^ ^22^, ^23^ ^]^ Subsequent side‐chain liberation using strong acid provided a flexible and convenient route for chemical synthesis of SICLOPPS‐derived peptides.

## 2 MATERIALS AND METHODS

The materials, methods, and compound characterization are described in detail in Appendix S1. Chemical reagents and solvents were manipulated using standard safety precautions in ventilated fume hoods.

## 3 RESULTS AND DISCUSSION

**SCHEME 1:**
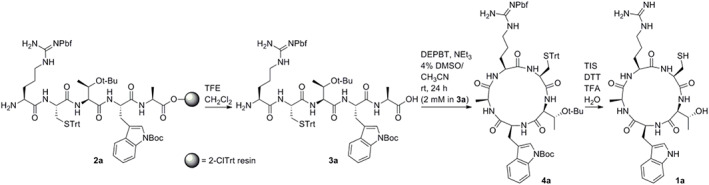
Synthesis of cyclic peptide **1a**

We chose four genetically‐encoded macrocycle library hits from our CPC screen for chemical synthesis: CTWAR, CKPIPTW, CPPNLLEL, and CIFKKSKP.^[^ ^18^ ^]^ We anticipated that the variable size of the targeted CPC hits, ranging from 5 to 8 residues, might impact the success of the macrocyclization step. The pentameric precursor (CTWAR) appeared most prone to epimerization and oligomerization reactions, which entail side‐products that are difficult to detect and remove.^[^ ^24^, ^25^ ^]^ To synthesize the CTWAR cyclopeptide **1a**, a RCTWA linear precursor was employed to minimize steric bulk at the extremities and mitigate side reactions. En route to cyclic pentapeptide **1a**, Fmoc/t‐Bu SPPS on 2‐ClTrt resin gave peptidyl resin **2a** (Scheme 1), which was cleaved using 30% 2,2,2‐trifluoroethanol (TFE) in dichloromethane to afford peptide **3a** bearing the anticipated 2,2,4,6,7‐pentamethyldihydrobenzofuran‐5‐sulfonyl (Pbf), trityl (Trt), tert‐butyl (t‐Bu), and benzyloxycarbonyl (Boc) protected side chains.^[^ ^26^, ^27^, ^28^ ^]^ After solvent removal, and in spite of the potential for trace fluorinated alcohol contaminant among other impurities, products **3** were found to be suitable for use in the next step without further purification.

Next, we probed the key synthetic transformation using a few milligrams (<5 μmol) of linear precursor **3a** and a reagent that has proven superior at avoiding epimerization, 3‐(diethoxyphosphoryloxy)‐1,2,3‐benzotriazin‐4(3H)‐one (DEPBT) (Scheme 1).^[^ ^29^ ^]^ During the macrolactamization reactions, we employed excess DEPBT (2–3 equivalents) and base [10 equivalents of triethylamine (TEA)] relative to linear precursors **3** to avoid the need for precise stoichiometry control. A reaction conducted in DMF at 2 mM concentration was monitored for progress by LC–MS analyses conduced at three time points (1, 4, and 13 h). Steadily declining substrate concentration was accompanied by the formation of macrocycle **4a**, which ultimately became the sole detectable species after 13–24 h. Integration of the UV signal at 220 nm gave a peak area of 39% relative to that initially produced by the linear starting material (Table 1, entry 1). The chosen analytical method thus failed to detect the remaining mass balance, which may be due to the formation of species which are insoluble or lack UV activity at 220 nm, such as polymers or deprotected side‐products. Use of DMSO as solvent led to a significantly improved outcome of 89% yield after 13 h (entry 2). Although the reaction failed in MeCN due to solubility (entry 3), DMSO/MeCN mixtures promoted high yields (80%–85%) that approached the value in pure DMSO. These DMSO/MeCN solvent mixtures were used in subsequent reactions for isolation of products; judicious selection of DMSO/MeCN mixtures avoided excessive amounts of high‐boiling point DMSO that would otherwise require removal by aqueous extraction.

**TABLE 1:** Solvent effects in **3a**–macrocyclization to **4a** (Scheme 1 conditions)

Entry	Solvent	% Yield over time^a^			
		1 h	4 h	13 h
1	DMF	11	21	39
2	DMSO	3	17	89
3	MeCN	—	—	—
4	DMSO/ MeCN (50: 50)	9	41	84
5	DMSO/ MeCN (20: 80)	9	25	85
6	DMSO/ MeCN (4: 96)	7	16	80

^a^ Determined by LC–MS.

With optimized macrocyclization conditions in hand, we undertook target synthesis on a larger scale. Macrocyclization reactions were conducted using 10–120 μmol of **3** in 8–170 ml of <1%–10% DMSO in MeCN. Completed reactions were quenched with acetic acid and readily reduced in volume by rotary evaporation to <2 ml due to the volatility of the MeCN component. Upon loading the crude residues **4** onto a preparatory C18 silica HPLC column, the polar injection solvent eluted at the solvent front, and side‐chain protected peptides **4** subsequently eluted in high purity with a mobile phase of high organic content (50%–95% MeOH, Table S1). Starting from linear **3a**, the process led to 16% isolated yield of **4a** (Table 2, entry a). To complete the synthesis, acidolytic liberation of the side‐chain protecting groups was achieved using a cocktail of triisopropyl silane, dithiothreitol, 2,2,2‐trifluoroacetic acid, and water to furnish cyclo‐[CTWAR] (**1a**) in 69% isolated yield.

**TABLE 2:** Transformation of linear peptides **3a**–**e** into cyclopentamers **1** and cyclodecameric side‐products **6** via side‐chain‐protected counterparts **4** and **5**, respectively
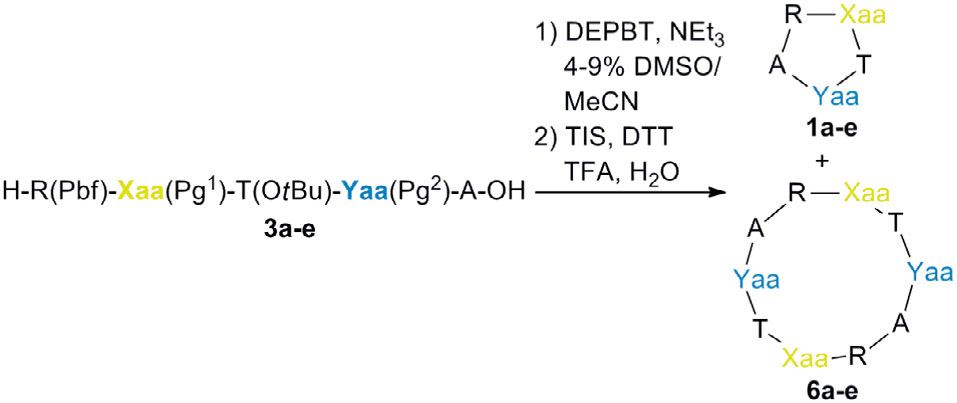

Entry	Xaa(Pg^1^) in 3	Yaa(Pg^2^) in 3	4 + 5^a^ yield (%)	1 + 6 yield (%)	1: 6 ratio^b^
**a**	Cys(Trt)	Trp(Boc)	16	69	>99: 1
**b**	Pro	Trp(Boc)	16	56	<1: 99
**c**	Ala	Trp(Boc)	27	65	86: 14
**d**	Cys(Trt)	Cys(Trt)	16	48	88: 12
**e**	Cys(Trt)	Ala	21	72	>99: 1

^a^ **5** = side‐chain protected **6**.

^b^ Estimated from nanoflow UHPLC‐HRMS.

The ease of cyclopentamer formation with target **1a** enabled us to explore analog synthesis (Table 2), the success of which critically depended on the **3**‐to‐**4** conversion. Using manual Fmoc/t‐Bu SPPS on 2‐ClTrt resin along with the TFE‐mediated cleavage, pentapeptides **3b**–**e** were obtained in 71%–95% yields and crude purities of 72%–92% (see Table S2). With the set of precursors in hand, we then investigated cyclopentamer scope.

Although linear pentapeptides **3b**–**e** contained minimal modification relative to parent **1a**, the macrocyclization‐deprotection sequence did not reliably furnish cyclopentapeptides **4** and their deprotected counterparts **1**. Variable amounts of cyclodecapeptides **5** were obtained during macrocyclization, as determined by mass spectrometric identification of the cognate deprotected derivatives **6**.

The linear precursor analog **3b** was generated by replacement of the Cys residue for Pro in RCTWA. Reaction of linear **3b** with DEPBT and TEA in DMSO (4%)/ MeCN followed by treatment with the cleavage cocktail and isolation gave 1.2 mg of product in 9% yield over two steps (Table 2, entry b). High‐resolution mass spectrometry (HRMS) characterization gave a 612.325 mass‐to‐charge ratio (*m*/*z*) corresponding to the anticipated [M + H]^+^ ion; however, the structure could not be unambiguously assigned due to uncertainty regarding the charge state (i.e., 2*m*/2*z* = *m*/*z*). An additional *m*/*z* 1223.642 signal corresponded either to a cyclodecamer **6b** [M' + H]^+^ ion, arising from a side reaction between two linear precursors, or a noncovalent [2M + H]^+^ **1b**‐adduct. Inspection of the lighter peak unveiled a 612.325/612.826/613.327 isotopic pattern of 0.5 dalton (Da) steps as opposed to the 1 Da progression within **1a** isotopes (i.e., m/z 618.281, 619.284, 620.287). The progressive 0.5 Da increases corresponded to a doubly‐charged [M' + 2H]^2+^ species and the isolated product was therefore not cyclopentamer **1b** but rather cyclo‐[PTWARPTWAR] (**6b**). With Ala as second residue in RCTWA (entry c), the reaction gave a cyclopentamer‐**1c** major component and cyclodecamer‐**6c** minor component in a ratio of 6:1. While default HPLC methods and instrumentation failed to separate the components to extract the latter ratio, sufficient resolution was achieved using nanoflow‐UHPLC‐HRMS (see Appendix S1). Substitution at the fourth Trp(Boc) residue for Cys(Trt) and Ala afforded a 6:1 cyclopentamer/cyclodecamer mixture and selective generation of **1e**, respectively (entries d and e). Overall, the synthesis of cyclopentameric **1** analogs was successful except for **1b**, which failed due to the presence of Pro.

Automated peptide synthesis was used to build linear precursors for six cyclic peptides **1** in the 7 to 8‐mer size range: cyclo‐[CKPIPTW] (**1f**), cyclo‐[CPPNLLEL] (**1h**), cyclo‐[CIFKKSKP] (**1j**), along with three scrambled variants (Table 3, Figure S1). The corresponding cyclizations were relatively robust due to reduced strain during the ring closure step, as well as the presence of at least one Pro in macrocyles **1f**–**k**. Within the larger sequences, a centrally‐located Pro promoted macrocyclization^[^ ^22^, ^23^, ^30^ ^]^; however, this proved insufficient to obtain target **1f** from syntheses using the CKPIPTW and KPIPTWC input sequences. Taking into consideration the impact of the bulky Cys(Trt) residue, tractable side‐chain protected macrocyclization precursors **3f**–**k** were obtained by a retrosynthetic disconnection strategy of preferentially situating both Pro and Cys(Trt) residues toward the linear precursor center. Fully automated SPPS of resin‐bound linear peptidyl ester intermediates **2f**–**k**, including the loading of the first amino acid, was achieved by modification of the synthesizer (CEM Liberty 1) default reaction times, temperatures, and protocols. TFE‐mediated cleavage yielded liberated linear peptides **3f**–**k** in 43%–60% yields and in 62%–98% crude purities (see Appendix S1). DEPBT‐activation generated the penultimate macrocycles **4f**–**k** in 12%–36% yields, while side chain liberation gave cyclopeptides **1f**–**k** in 32%–49% yields with an absence of detectable cyclotetradecamer and cyclohexadecamer side products. Detection of [2M − 1]^+^ ions was attributed to adventitious disulfide formation and addressed by addition of tris(2‐carboxyethyl)phosphine (TCEP, 50 mM) as a reducing agent to the analytical samples prior to injection during LC–MS. In summary, successful syntheses of the 7/8‐mer cyclic peptide targets **1f**–**k** were enabled by optimization of the linear precursors **3f**–**k**.

**TABLE 3:** Sequence scope during the synthesis of cycloheptamers and cyclooctamers **1f**–**k**

Entry	Sequence	3^a^ yield (%)	4^a^ yield (%)	1 yield (%)
**f**	WCKPIPT	50	12	39
**g**	KCKPFKSI	44	22	44
**h**	ELCPPNLL	47	26	32
**i**	TKPCPWI	44	29^b^	41
**j**	KSKPCIFK	60	36	32
**k**	LEPLNPLC	43	23	49

*Note*: Synthetic route analogous to Scheme 1.

^a^ Canonical Fmoc/tBu side chain protecting groups were employed in intermediates **3**–**4**, see the Appendix S1 for details.

^b^ 0.6 mM in **3i** with 5% DMF in MeCN as solvent.

During efforts to rapidly iterate cyclic peptide analog synthesis and testing, throughput was prioritized by application of standardized reaction conditions. Our standardized conditions successfully delivered the intended targets and analogs for 7‐ and 8‐mer cyclic peptides; however, the synthesis of 5‐mer cyclic peptides proved less predictable due to the competitive formation of cyclodecamers, which themselves may also be of utility as biological probes due to potential avidity effects and/or other properties. Moreover, the confounding effects of such oligomers can be readily avoided by application of nanoflow‐UHPLC‐HRMS in conjunction with isotopic‐pattern analysis, as described herein.

**FIGURE 1:**
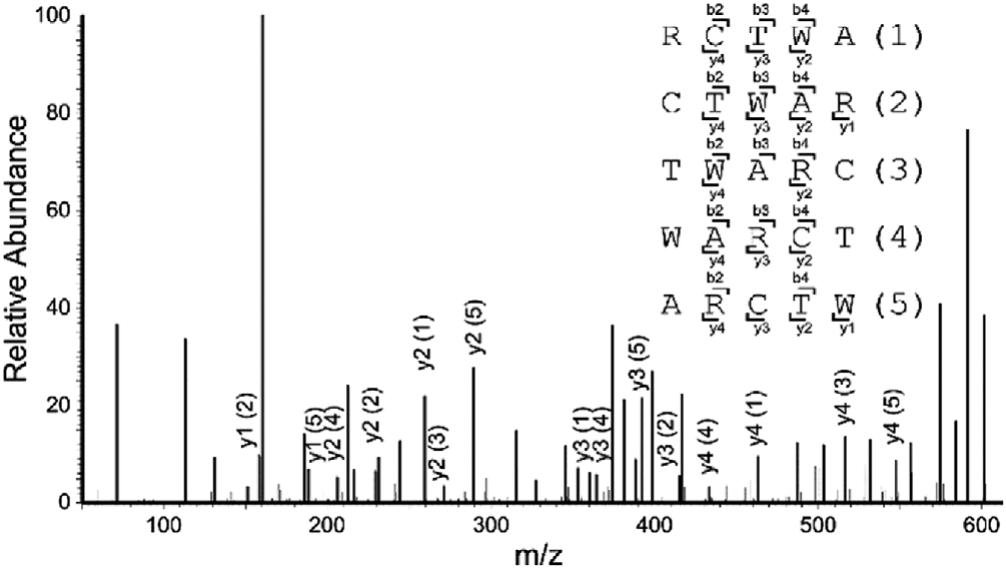
HCD MS/MS fragmentation spectrum of **1a**. Annotations for selected y peptide fragment ions are shown. Standard peptidic fragment ion nomenclature^[^ ^33^ ^]^ was adapted by using the illustrated arbitrary 1–5 numerical assignments for the isomeric ring‐opening intermediates

In addition to characterization of peptides **1** and **6** by exact mass, we employed a MS fragmentation (MS^*2*^) method to obtain explicit sequence confirmation by way of higher‐energy collisional dissociation tandem mass spectrometry (HCD MS/MS, Table S3). As representative example, the MS/MS spectrum of **1a** illustrates the dozens of fragment ions that were generated (Figure 1). Cyclic peptides undergo quasi‐statistical ring opening to afford several linear intermediate ions, ultimately producing many more fragments compared to their linear peptide counterparts.^[^ ^31^ ^]^ The mMass software package was used to annotate MS/MS fragments and confirm peptidic sequences.^[^ ^32^ ^]^ We observed a large proportion of the potential b and y **1**‐fragments (45%–91% of 35, 77, or 104), allowing for high sequence confirmation confidence even in additional fully blinded experiments. In the context of combinatorial SICLOPPS library hits, DNA sequence analysis of the genetically‐encoded peptide is used to infer cyclic peptide structure. MS^*n*^ analysis provides the additional capacity to decipher post‐translational modifications that may be introduced *in vivo* or chemical modifications introduced during synthesis *in vitro*.

Chromosomal missegregation in HeLa cells was assayed by incubation for 41 h with 1–25 μM of cyclic peptides **1**, fixation, and fluorescence staining to visualize tubulin, DNA, and Aurora B kinase. However, we failed to detect predicted mitotic defects such as multinucleation and micronucleation (data not shown). The lack of activity in this assay may be explained by the fact that the genetically‐encoded hits from our previous SICLOPPS screen were modest,^[^ ^18^ ^]^ and that exogenous introduction of peptides **1** to cells involves overcoming well‐known peptide permeability barriers.

## 4 CONCLUSIONS

A series of cyclic peptides **1** were chemically synthesized and characterized by mass spectrometry through a combination of exact mass, isotopic pattern, and collision‐induced fragmentation. Application of the latter techniques serves to increase structural assignment confidence. In particular, isotopic pattern analysis allowed cyclic peptides **1** to be distinguished from side products that, in spite of their doubled molecular weight, displayed the targeted MS signal due to a predisposition toward doubly‐charged states. Collision‐induced fragmentation was used to explicitly observe the sequence of **1**, a method that would also facilitate the identification cyclic peptide products in complex biological samples. The chosen sequences were designed to recapitulate genetically‐encoded hits that were previously identified in a SICLOPPS screen for cyclic peptides that target the CPC.^[^ ^18^ ^]^ The synthesized versions matched the genetically‐encoded counterparts both in the variable portion as well as the constant cysteine residue. We note that in the context of chemical synthesis, Cys residues provide a useful handle for diversification by selective modifications,^[^ ^34^ ^]^ including by cell‐penetrating TAT peptides and by fluorescent probes.^[^ ^35^, ^36^, ^37^ ^]^ Automated linear peptide synthesis followed by ring closure as described here will enable the routine validation of cyclic peptides identified as hits in diverse genetically‐encoded SICLOPPS library screens.

## CONFLICT OF INTEREST

There are no conflicts of interest to declare.

## Supporting information


**Appendix** **S1**: Synthetic procedures, characterization, and spectra.

## Data Availability

All data needed to evaluate the conclusions in the paper are present in the paper and/or in the Appendix S1.
