# Supplementary material for: Head‐to‐tail cyclization of side chain‐protected linear peptides to recapitulate genetically‐encoded cyclized peptides
Source: Pept Sci (Hoboken). 2022 Jan 6;114(3):e24254. doi: 10.1002/pep2.24254 (PMC9286623; doi:10.1002/pep2.24254)
Supplement: Supplementary file 1 — Appendix S1: Synthetic procedures, characterization, and spectra. [file PEP2-114-0-s001.pdf]

Head-to-tail cyclization of side chain-protected linear peptides to recapitulate genetically-encoded cyclized peptides

Samir Bouayad-Gervais, Daniel J. St-Cyr, Mathieu Courcelles, Éric Bonneil,
Florence H. Gohard, Pierre Thibault, William C. Earnshaw and Mike Tyers

Appendix S1

Table of Contents

I. General Procedures 3
Materials 3
Manual peptide synthesis 3
Manual Trt resin loading procedure 3
Manual peptide elongation procedure 4
Automated peptide synthesis 4
Automated Trt resin loading procedure 5
Automated peptide elongation procedure. 6
TFE-mediated Trt resin cleavage procedure 6
Peptide macrocyclization procedure 6
TFA-mediated deprotection procedure 7
Chromatographic characterization of peptidic products 7
II. Synthesis and characterization of cyclic peptides 1 and 6 10
Synthesis of cyclic RCTWA 10
Intermediate 3a: H-Arg(Pbf)-Cys(Trt)-Thr(t-Bu)-Trp(Boc)-Ala-OH 10
Intermediate 4a: *cyclo*-[Arg(Pbf)-Cys(Trt)-Thr(t-Bu)-Trp(Boc)-Ala] 11
Product 1a: *cyclo*-[Arg-Cys-Thr-Trp-Ala] 11
Attempted synthesis of cyclic RPTWA 13
Intermediate 3b: H-Arg(Pbf)-Pro-Thr(t-Bu)-Trp(Boc)-Ala-OH 13
Intermediate 5b: cyclo-[(Arg(pfb)-Pro-Thr(t-Bu)-Trp(Boc)-Ala)$_2$] 14
Side-product 6b: cyclo-[(Arg-Pro-Thr-Trp-Ala)$_2$] 15
Synthesis of cyclic RATWA 16
Intermediate 3c: H-Arg(Pbf)-Ala-Thr(t-Bu)-Trp(Boc)-Ala-OH 16
Intermediates 4c and 5c: cyclo-[Arg(Pbf)-Ala-Thr(t-Bu)-Trp(Boc)-Ala] with dimer 17
Product 1c/ side-product 6c mixture: cyclo-[Arg-Ala-Thr-Trp-Ala] with dimer 18
Synthesis of cyclic RCTCA 20
Intermediate 3d: H-Arg(Pbf)-Pro-Thr(t-Bu)-Cys(Trt)-Ala-OH 20
Intermediate 4d and 5d: cyclo-[Arg(Pbf)-Cys(Trt)-Thr(t-Bu)-Cys(Trt)-Ala] with dimer 21
Product 1d/ side-product 6d mixture: cyclo-[Arg-Cys-Thr-Cys-Ala] with dimer 22

Synthesis of cyclic RCTAA 24
Intermediate 3e: H-Arg(Pbf)-Cys(Trt)-Thr(t-Bu)-Ala-Ala-OH 24
Intermediate 4e: cyclo-[Arg(Pbf)-Cys(Trt)-Thr(t-Bu)-Ala-Ala] 24
Product 1e: cyclo-[Arg-Cys-Thr-Ala-Ala] 25
Synthesis of cyclic WCKPIPT 26
Intermediate 3f: H-Trp(Boc)-Cys(Trt)-Lys(Boc)-Pro-Ile-Pro-Thr(O-tBu)-OH 26
Intermediate 4f: cyclo-[Trp(Boc)-Cys(Trt)-Lys(Boc)-Pro-Ile-Pro-Thr(O-tBu)] 27
Product 1f: cyclo-[Trp-Cys-Lys-Pro-Ile-Pro-Thr] 27
Synthesis of cyclic KCKPFKSI 29
Intermediate 3g: H-Lys(Boc)-Cys(Trt)-Lys(Boc)-Pro-Phe-Lys(Boc)-Ser(t-Bu)-Ile-OH 29
Intermediate 4g: cyclo-[Lys(Boc)-Cys(Trt)-Lys(Boc)-Pro-Phe-Lys(Boc)-Ser(t-Bu)-Ile] 30
Product 1g: cyclo-[Lys-Cys-Lys-Pro-Phe-Lys-Ser-Ile] 31
Synthesis of cyclic ELCPPNLL 32
Intermediate 3h: H-Glu(t-Bu)-Leu-Cys(Trt)-Pro-Pro-Asn(Trt)-Leu-Leu-OH 32
Intermediate 4h: cyclo-[Glu(O-tBu)-Leu-Cys(Trt)-Pro-Pro-Asn(Trt)-Leu-Leu] 33
Product 1h: cyclo-[Glu-Leu-Cys-Pro-Pro-Asn-Leu-Leu] 33
Synthesis of cyclic TKPCPWI 34
Intermediate 3i: H-Thr(t-Bu)-Lys(Boc)-Pro-Cys(Trt)-Pro-Trp(Boc)-Ile-OH 34
Intermediate 4i: cyclo-[Thr(t-Bu)-Lys(Boc)-Pro-Cys(Trt)-Pro-Trp(Boc)-Ile] 35
Product 1i: cyclo-[Thr-Lys-Pro-Cys-Pro-Trp-Ile] 36
Synthesis of cyclic KSKPCIFK 37
Intermediate 3j: H-Lys(Boc)-Ser(t-Bu)-Lys(Boc)-Pro-Cys(Trt)-Ile-Phe-Lys(Boc)-OH 37
Intermediate 4j: cyclo-[Lys(Boc)-Ser(t-Bu)-Lys(Boc)-Pro-Cys(Trt)-Ile-Phe-Lys(Boc)] 38
Product 1j: cyclo-[Lys-Ser-Lys-Pro-Cys-Ile-Phe-Lys] 38
Synthesis of cyclic LEPLNPLC 40
Intermediate 3k: H-Leu-Glu(t-Bu)-Pro-Leu-Asn(Trt)-Pro-Leu-Cys(Trt)-OH 40
Intermediate 4k: cyclo-[Leu-Glu(t-Bu)-Pro-Asn(Trt)-Pro-Leu-Cys(Trt)] 40
Product 1k: cyclo-[Leu-Glu-Pro-Leu-Asn-Pro-Leu-Cys] 41
III. References 42

I. General Procedures

Materials

Fluorenylmethyloxycarbonyl (Fmoc) *N*-protected amino acids were purchased from CEM Inc. (Matthews, NC). The following chemicals were obtained from Sigma Aldrich Inc. (St. Louis, MO): 3-(diethoxyphosphoryloxy)-1,2,3-benzotriazin-4(3H)-one (DEPBT), (7-azabenzotriazol-1-yl)-N,N,N',N'-tetramethyluronium hexafluorophosphate (HATU), benzotriazole-1-yl-N,N,N',N'-tetramethyluronium hexafluorophosphate (HBTU), dithiothreitol (DTT) and 2,2,2-trifluoroethanol (TFE). Tris(2-carboxyethyl)phosphine (TCEP) was obtained from Pierce Biotechnology (Rockford, IL). The following chemicals were obtained from Fischer Scientific Inc. (Waltham, MA): dimethyl formamide (DMF), dimethylsulfoxide (DMSO), acetonitrile (MeCN), and dichloromethane (CH_2Cl_2). Anhydrous DMF and CH_2Cl_2 were prepared by treating the solvents with activated molecular sieves (4 Å, ca. 20% by volume) under nitrogen and waiting >2 d prior to use. Chloro-(2'-chloro)trityl polystyrene (2-Cl-Trt-Cl) resin was purchased from Rapp Polymere GmbH (Tüebingen, Germany). The preloaded resin **7e** [H-Lys(Boc)-2-Chlorotrityl resin, styrene with 1% divinylbenzene copolymer, 100-200 mesh, 0.54 mmol/g] was bought from GL Biochem Ltd. (Shanghai, China). Unless specified otherwise chemicals were used without further purification.

Manual peptide synthesis

During manual peptide elongation, the highly-reactive HATU amide bond coupling reagent was chosen to reduce reaction times and avoid resort to repeated couplings. To facilitate the optimal use of HATU under anhydrous conditions, resin for manual SPPS was loaded into disposable polypropylene syringes fitted with polypropylene frits (70 μm porosity) and needles rather than filter tubes.[1] To remove adventitious water from resin-charged fritted syringes, dry solvents (DMF or CH_2Cl_2) under nitrogen were repeatedly (2–5 times) aspirated into and expelled from the syringe over 5–30 minutes.

Manual Trt resin loading procedure

2-ClTrt-Cl Resin (250 mg, 0.250 mmol, 1 equiv.) was loaded into a fritted syringe (6 mL capacity), washed twice with dry CH_2Cl_2, and immersed in dry DMF under nitrogen. After 0.5 h, the liquid phase was expelled and the solid phase was treated with a mixture of Fmoc amino acid (0.3 mmol, 1.2 equiv.) and Hünig's base (218 μL, 1.25 mmol, 5 equiv.) in CH_2Cl_2 (2.5 mL). After mixing by repeated inversion at room temperature

for 2 h, the reaction medium was removed and the resin was washed multiple times with CH_2Cl_2/ MeOH/ Hünig's base (17:2:1, 3x over 3 min), CH_2Cl_2 (3x over 3 min), DMF (2x over 2 min), and CH_2Cl_2 (2x over 2 min). The resulting 2-ClTrt resin bearing a single Fmoc amino acid was used directly in subsequent elongation reactions by assuming quantitative yield (0.25 mmol) for subsequent stoichiometry calculations.

Manual peptide elongation procedure

2-ClTrt resin bearing an *N*-terminal Fmoc amino acid residue (0.25 mmol nominal loading) was deprotected by treatment with two batches of piperidine (20%) in DMF (3-4 mL) over a total of 17 min (2 min, then 15 min) and the liquid phase was removed. The solid phase was washed multiple times with DMF (5x over 5 min) and CH_2Cl_2 (2x over 2 min) to afford a resin-bound free amine. The latter was elongated by treatment with a dry DMF (2.8–3 mL) solution of the subsequent Fmoc-amino acid (0.750 mmol, 3 equiv.), HATU (271 mg, 0.713 mmol, 2.85 equiv.), and Hünig's base (258 µL, 1.48 mmol, 5.9 equiv.). After mixing by repeated inversion at room temperature for 45 min, the reaction medium was removed and the resin was washed multiple times with DMF (5x over 5 min) and CH_2Cl_2 (2x over 2 min). To confirm reaction completion, resin aliquots from before and after the acylation reaction were subjected to the Kaiser colorimetric test and compared.[2] The deprotection/elongation sequence was appropriately iterated according to the targeted sequence to afford an Fmoc-peptidyl resin, which was deprotected by repeating the piperidine process to afford the final *N*-terminal free amine peptidyl resin **2**. The latter was used directly in the TFE-mediated Trt resin cleavage procedure described below.

Automated peptide synthesis

Automated Solid Phase Peptide Synthesis (SPPS) was conducted on a CEM Liberty1 microwave synthesizer, which accelerates peptide synthesis on Rink amide and Wang resins by employing microwave heating to 75 °C during coupling/deprotection cycles.[3] Microwave-accelerated peptide synthesis on 2-Cl-Trt-Cl resin preferentially occurs at 50 °C,[4, 5] which was modified as elaborated below to accommodate automated attachment of the first amino acid. During automated peptide synthesis, complications due to frit clogging were overcome by substituting resins featuring standard bead diameter ranges [100-200 mesh (74–149 µm)] with larger counterparts (125–160 µm or 250–315 µm).

Automated Trt resin loading procedure

A Liberty1 synthesizer was charged with 2-ClTrt-Cl resin (250 mg, 1.0 mmol/g, 0.25 mmol), 35% Hünig's base in DMF (1.5 mL, ca. 3.9 mmol, steps 1–2, Figure S1), and Fmoc amino acid in CH_2Cl_2 (3.75 mL, 0.2 M, 0.75 mmol, step 3). The mixture was heated to 50 °C by microwave irradiation for 30 min, the liquid phase was removed (step 4), and the solid phase was washed with DMF (20 mL total, steps 5–6). The coupling and wash sequence was repeated (steps 7–12), and the resulting Fmoc amino acid resin was treated with 35% Hünig's base in DMF (4 mL, ca. 10 mmol, step 13) and MeOH (3.75 mL, 93 mmol, step 14). After mixing by argon bubbling for 10 min at room temperature (step 15), the liquid phase was removed and the resin was washed with DMF (10 mL, step 16). The capping sequence was repeated (steps 17–20) and the resin was washed with additional DMF (20 mL total, steps 21–22) to afford 2-ClTrt resin bearing a single Fmoc amino acid. The latter was used directly in subsequent elongation reactions by assuming quantitative yield (0.25 mmol) for subsequent stoichiometry calculations.

Figure S1. Screenshot of the automated method used for loading Fmoc-amino acids onto 2-ClTrt-Cl resin using a Liberty1 instrument.

	Operation	Parameter	Volume	Drain	Cycles	Pause
1	Wash - Top	Main Wash (DMF)	10	☑	1	☐
2	Add Activator Base	DIEA	1.5	☐	1	☐
3	Add Amino Acid		3.75	☐	1	☐
4	Microwave Method	Coupling 50C trit		☑	1	☐
5	Wash - Top	Main Wash (DMF)	10	☑	1	☐
6	Wash - Bottom	Main Wash (DMF)	10	☑	1	☐
7	Add Activator Base	DIEA	1.5	☐	1	☐
8	Add Amino Acid		3.75	☐	1	☐
9	Microwave Method	Coupling 50C trit		☑	1	☐
10	Wash - Top	Main Wash (DMF)	10	☑	1	☐
11	Wash - Bottom	Main Wash (DMF)	10	☑	1	☐
12	Wash - Top	Main Wash (DMF)	10	☑	1	☐
13	Add Activator Base	DIEA	4	☐	1	☐
14	Add Custom Amino Acid	Position 23 (EX3 - 3)	3.75	☐	1	☐
15	Wait State		600	☐	1	☐
16	Wash - Top	Main Wash (DMF)	10	☑	1	☐
17	Add Activator Base	DIEA	4	☐	1	☐
18	Add Custom Amino Acid	Position 23 (EX3 - 3)	3.75	☐	1	☐
19	Wait State		600	☐	1	☐
20	Wash - Top	Main Wash (DMF)	10	☑	1	☐
21	Wash - Bottom	Main Wash (DMF)	10	☑	1	☐
22	Wash - Top	Main Wash (DMF)	10	☑	1	☐

Automated peptide elongation procedure.

Resin-bound amino acid was achieved using the recommended precursors, reagents, and solvents, including DMF (peptide grade) solutions of Fmoc-amino acids (0.2 M), HBTU (0.5 M), Hünigs base (35%), and piperidine (20%).[3] The default microwave heating setting for the coupling was changed from 75 °C to 50 °C and the default 300 s coupling reaction time setting was changed to 1800 s. As final automated step, the resin was treated with piperidine (20%) in DMF, heated to 50 °C for 30 min, and washed with DMF to afford *N*-terminal free amine peptidyl resin **2**. The latter was transferred from the synthesizer into a fritted syringe for direct use in the TFE-mediated resin cleavage procedure.

TFE-mediated Trt resin cleavage procedure

N-terminal free amine peptidyl resin **2** was treated with TFE (30%) in CH_2Cl_2 (12 mL), mixed by periodic inversion for 1 h at room temperature, and the liquid phase was collected. The cleavage was repeated using fresh TFE/ CH_2Cl_2 mixture and the combined liquid phases were concentrated *in vacuo*. The resulting crude side-chain protected peptide **3** was employed directly in the linear peptide macrocyclization procedure.

Peptide macrocyclization procedure

A solution of NEt_3 (35 µL, 250 µmol, 10 equiv.) in CH_3CN (11.2 mL) was added to a DMSO (470 µL) solution of linear peptide **3** (25 µmol, 1 equiv.) and the resulting mixture was treated with DEPBT (18.6 mg, 62 µmol, 2.5 equiv.). After stirring at room temperature for 24 h, the reaction was quenched with AcOH (0.5–1 mL) and the volume was reduced *in vacuo* by ≥ 10 fold (to 1–2 mL). The resulting DMSO/CH_3CN solution of the crude was filtered (3 mm syringe filter, 0.2 µm pore size) and purified by preparatory HPLC to afford cyclic peptides **4**, **5**, or a **4**/**5** mixture dependent on sequence. Preparatory HPLC purifications were performed on an Agilent 1200 instrument or a Waters Inc. (Milford, MA) 2795 coupled to a 2996 diode array and micromass ZQ for UV and MS detection respectively. Cyclic peptides **4** were eluted using flow rates of 20 mL/min under the conditions detailed in Table S1. The collected fractions were concentrated *in vacuo* (2–5 Torr) at 50 °C, then concentrated to dryness with assistance from multiple azeotropic coevaporations with *i*-PrOH or 1,4-dioxane as necessary.

Table S1. Methods used for preparatory HPLC purification.

#	Column	Eluent system (weak mixture "A"/ strong mixture "B")	Gradient	Peak detection
A	Kinetex C18 100A AXIA 21.2 x 100 mm	[H_2O / MeOH / TFA (95 : 5 : 0.1)] / [MeOH / H_2O / TFA (95 : 5 : 0.1)]	0-3 min, 70% "B" in "A"; 3-10 min, 70 to 100% "B": 10-20 min 100% "B".	UV: 220.16, 254.16.
B	Zorbax SB-C18 PrepHT 5 µm; 21.2 x 100 mm	[H_2O / MeOH / AcOH (95 : 5 : 0.1)] / [MeOH / H_2O / AcOH (95 : 5 : 0.1)]	0-2 min, 20% "B" in "A"; 2-15 min, 20 to 100% "B"; 15-20 min, 100% "B".	UV: 220.4 nm, 254.4 nm. or MS:
C	Zorbax SB-C18 PrepHT 5 µm; 21.2 x 100 mm	[H_2O / MeOH / TFA (95 : 5 : 0.05)] / [MeOH / H_2O / TFA (95 : 5 : 0.05)]	0-2 min, 50% "B" in "A"; 2-10 min, 50 to 100% "B"; 10-15 min, 100% "B".	UV: 220.4 nm, 254.4 nm.
D	Atlantis Prep. OBD, 5 µm: 30 x 100 mm	[H_2O/ MeOH/ HCO_2H (95 : 5 : 0.1)] / [MeOH/ H_2O/ HCO_2H (95 : 5 : 0.1)]	0-15 min, 20 to 75% "B" in "A".	MS, ESI+

TFA-mediated deprotection procedure

To avoid known complications associated with the deprotection of peptides containing Cys(Trt) and Trp(Boc) residues, the cleavage cocktail containing DTT as nucleophilic scavenger was employed.[6] Among alternative thiols, DTT was selected due to reduced stench.

A heterogeneous mixture of DTT (~0.6 mg, 4 µmol), H_2O (30 µl), TFA (0.53 mL), and triisopropylsilane (12.1 µl, 59 µmol) was added to protected peptide **4**, **5**, or a **4**/**5** mixture (4 µmol) and stirred for 0.5 h at room temperature. The volatiles were removed in vacuo and an *i*-PrOH solution of the residue was purified by manual reversed-phase chromatography using a Sep-Pak C-18 3cc cartridge (Waters Inc., 37–55µm size) and 20% MeOH in H_2O as eluent when crude peptides were obtained in low quantities (< 7 mg). Otherwise, purification was conducted with DMSO solutions using preparative HPLC. The collected fractions were concentrated *in vacuo* (1–5 Torr) at 50 °C, then concentrated to dryness with assistance from multiple azeotropic coevaporations with *i*-PrOH or 1,4-dioxane. The residue was dissolved in dilute $HCl_{(aq)}$ (> 10 equiv.), and lyophilized to yield **1**, **6**, or a **1**/**6** mixture, typically as HCl salts, dependent on structure.

Chromatographic characterization of peptidic products

The purity and identity of synthetic intermediates **3**–**5** was established by a combination of Analytical High Performance Liquid Chromatography (analytical HPLC), HPLC-low resolution Mass Spectrometry (HPLC-MS), and HPLC-High Resolution Mass Spectrometry (HPLC-HRMS). Analytical HPLC was monitored by diode array UV detector and a 1260 infinity Evaporative Light Scattering (ELS) detector

operating at 50 °C. Analytical HPLC-MS spectra were recorded on an Agilent Inc. (Santa Clara, CA) 1200 series HPLC coupled to a diode array UV detector and a 6120 Quadrupole low resolution mass spectrometer equipped with an Electrospray Ionization (ESI) source. HPLC-HRMS spectra were recorded using an Agilent Mass Selective Detector with Time-of-Flight analyzer (MSD-TOF, model 61969A). Analytes were eluted under the conditions listed in Table S2.

Cyclic peptides **1** and **6** were analyzed using a Dionex/Thermo UltiMate 3000 binary RSLCnano Ultra High Performance Liquid Chromatography (UHPLC) system coupled to a Q-Exactive MS operating under the chromatographic and spectrometric conditions detailed in Table S2 and Table S3. Purified cyclic peptides **1** harbouring Cys residues were prone to dimerization by disulfide bond formation, complicating mass spectrometry analyses, which was avoided by adding TCEP (0.5 mM final concentration) reductant to samples prior to injection.

Table S2. Methods used for analytical HPLC-MS, HPLC-ELS, and UHPLC-HRMS analysis.

#	Column	Flow (mL/min)	Eluent system (weak mixture "A"/ strong mixture "B")	Gradient	Peak detection
**LC-MS**					
E	Kinetex-C18, 2.6 µm; 3.0 x 30 mm	1.50	[H_2O / MeOH / AcOH (95 : 5 : 0.1)] / [MeOH / H_2O / AcOH (95 : 5 : 0.1)]	0-0.5 min, 0% to 100% "B" in "A"; 0.5-2 min, 100% "B".	UV: 220.4 nm, 254.4 nm. MS: ESI+, ESI-
F	Kinetex-C18, 2.6 µm; 3.0 x 30 mm	1.0	[H_2O/ MeOH/ HCO_2H (95: 5 : 0.1)] / [MeOH/ H_2O/ HCO_2H (95 : 5 : 0.1)]	0 to ~7 min, 70 to 80% "B" in "A"; ~7 to 15 min, 80% "B".	UV: 220.4 nm, 254.4 nm. MS: ESI+, ESI-
G	Agilent Poroshell 120; EC-C18, 2.7 µm; 2.1 x 30 mm	1.0	[H_2O / MeOH / AcOH (95 : 5 : 0.1)] / [MeOH / H_2O / AcOH (95 : 5 : 0.1)]	0-1.5 min, 0% to 100% "B" in "A"; 1.5-4 min, 100% "B".	UV: 220.4 nm, 254.4 nm. MS: ESI+, ESI-
**HPLC-ELS**					
H	Zorbax SB-Phenyl 3.5 µm; 4.6 x 30mm.	1.0	[H_2O / MeOH / TFA (95 : 5 : 0.05)] / [MeOH / H_2O / TFA (95 : 5 : 0.05)]	0-1.5 min, 0% to 100% "B" in "A"; 1.5-4 min, 100% "B".	UV: 220.8 nm, 254.8 nm. ELS: 50 °C
**UHPLC-HRMS**					
I	Phenomenex Jupiter C18, 3µm, 300 Å, 15 cm x 150 µm[a]	600 nL/ min	[H_2O / HCO_2H (99.8 :0.2)] / [MeCN / HCO_2H (99.8 :0.2)]	0-30 min, 50 to 99% "B" in "A".	HRMS: ESI+
J	Phenomenex Jupiter C18, 3µm, 300 Å, 15 cm x 150 µm[a]	600 nL/ min	[H_2O / HCO_2H (99.8 :0.2)] / [MeCN / HCO_2H (99.8 :0.2)]	0-70 min, 40 to 99% "B" in "A".	HRMS: ESI+

[a] Trap column: Phenomenex Jupiter C18, 3µm, 300 Å, 0.5 cm x 360 µm.

Table S3. MS and MS/MS Parameters used during UHPLC analysis.

**Parameter**	**Value**
Instrument	Q-Exactive
Run time	30 or 70 min
Spray voltage	+ 3.5 kV
MS1 scan range	300-1500 m/z
MS1 resolution	70 000
MS1 AGC target	1e6
MS1 injection time	100 ms
MS2 resolution	35 000
MS2 ACG target	5e5
MS2 injection time	500 ms
MS2 Isolation window	2.0 m/z
MS2 HCD - Normalized collision energy	27
MS2 intensity threshold	1e4
MS2 dynamic exclusion	10 s
MS2 inclusion list	yes

II. Synthesis and characterization of cyclic peptides 1 and 6

Synthesis of cyclic RCTWA

Intermediate 3a: H-Arg(Pbf)-Cys(Trt)-Thr(t-Bu)-Trp(Boc)-Ala-OH

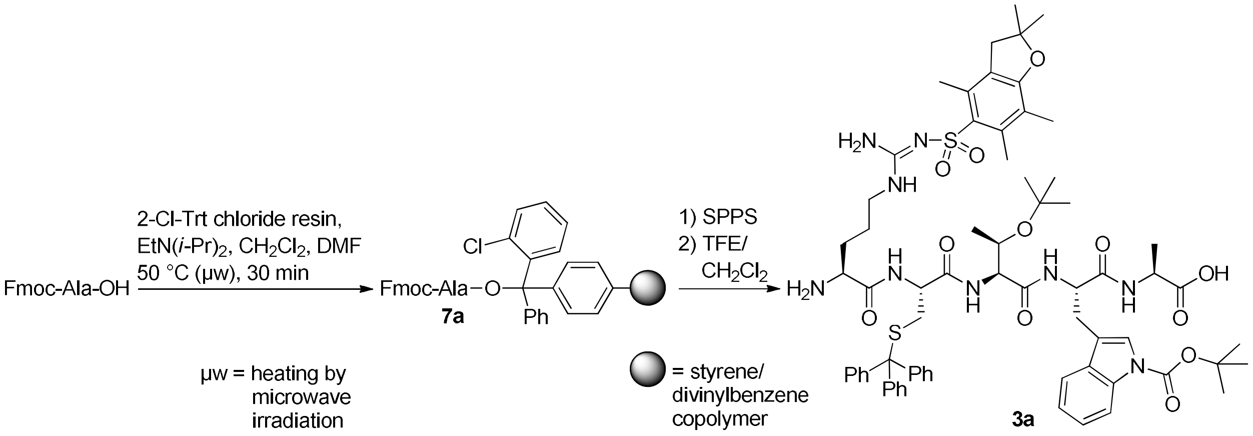

The automated Trt resin loading procedure was executed using a CH_2Cl_2 solution of Fmoc-Ala-OH (5 mL, 0.2 M, 1 mmol) and 2.75 mL of MeOH during resin capping to afford Fmoc-alaninyl Trt resin **7a**. Continuation of SPPS by the automated peptide elongation procedure using a more dilute HBTU solution (0.5 M) and standard DMF solutions (0.2 M) of Fmoc-Trp(Boc)-OH, Fmoc-Thr(*t*-Bu)-OH, Fmoc-Cys(Trt)-OH, and Fmoc-Arg(Pbf)-OH as inputs ultimately afforded *N*-terminal free amine peptidyl resin **2a**. The latter was subjected to the TFE-mediated Trt resin cleavage procedure to afford 144 mg (MW 1286.60, 112 µmol, 45% yield) of side chain-protected peptide **3a** as an off-white powder. HPLC-MS characterization using method "E"; retention time: 1.51 min, crude purity: 53 %, MS (ESI$^+$) *m*/*z*: $[M+H]^+$ Calcd for $C_{68}H_{87}N_9O_{12}S_2$ 1286.6, found 1286.4

Intermediate 4a: *cyclo*-[Arg(Pbf)-Cys(Trt)-Thr(t-Bu)-Trp(Boc)-Ala]

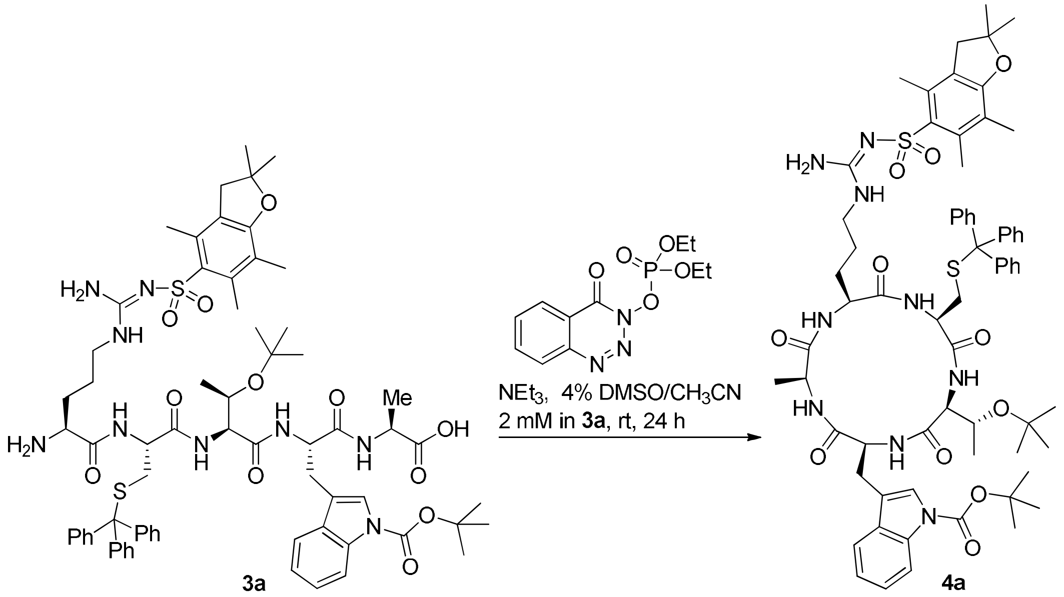

Side chain-protected peptide **3a** (32 mg, 25 µmol) was subjected to the peptide macrocyclization procedure using preparatory HPLC method "A" for purification to ultimately afford 5 mg (MW 1268.59, 3.9 µmol, 16% yield) protected cyclic peptide **4a** as a white solid. HPLC-MS characterization using method "E"; retention time: 1.97 min, purity: 98 %, MS (ESI$^+$) *m*/*z*: $[M+H–Boc]^+$ Calcd for $C_{63}H_{78}N_9O_9S_2$ 1168.5, found 1169.0.

Product 1a: *cyclo*-[Arg-Cys-Thr-Trp-Ala]

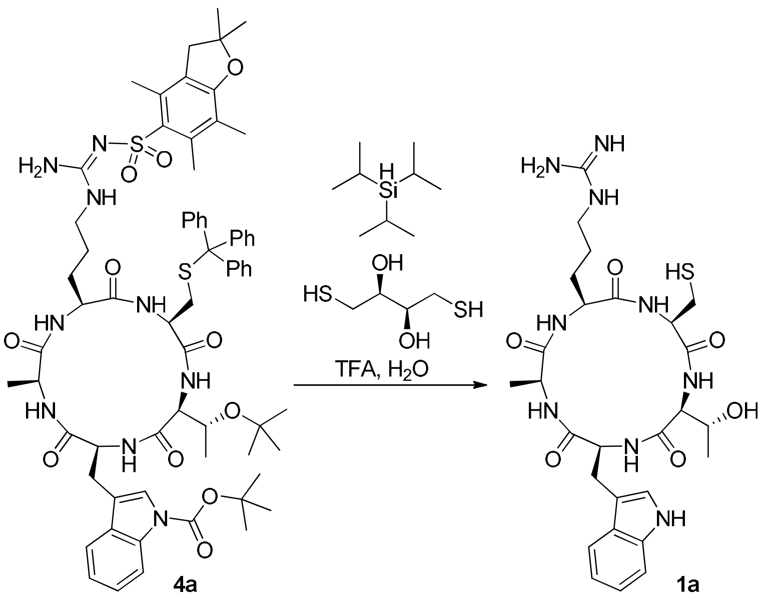

The TFA-mediated deprotection procedure was conducted on peptide **4a** (5 mg, 3.9 µmol) using manual preparative HPLC-MS in conjuction with eluent system 20% MeOH in Water for purification to ultimately afford 2 mg (TFA salt FW: 731.7, 2.7 µmol, 69% yield) of *cyclo*-[argininyl-cysteinyl-threoninyl-tryptophanyl-alanine] (**1a**) as a solid. UHPLC-HRMS characterization using method "I"; retention time: 13.3 min, HRMS (ESI$^+$) *m*/*z*: $[M+H]^+$ Calcd for $C_{27}H_{40}N_9O_6S$ 618.2817, found 618.2811. HCD MS/MS fragment count: Calcd for b/y ions 35, found 30.

Mass chromatogram of **1a** (Total Ion Chromatogram, TIC)

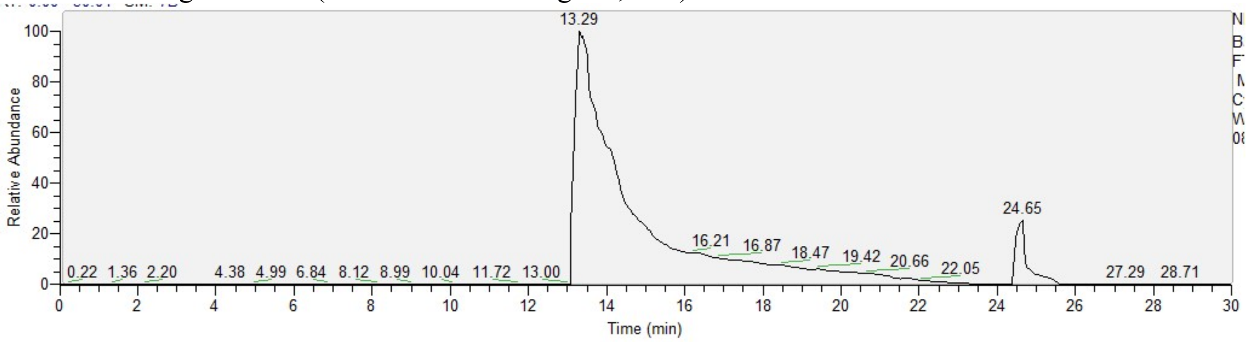

MS expansion illustrating the isotopic profile of **1a**. The resolution (R) and charge state (Z) of each peak is given.

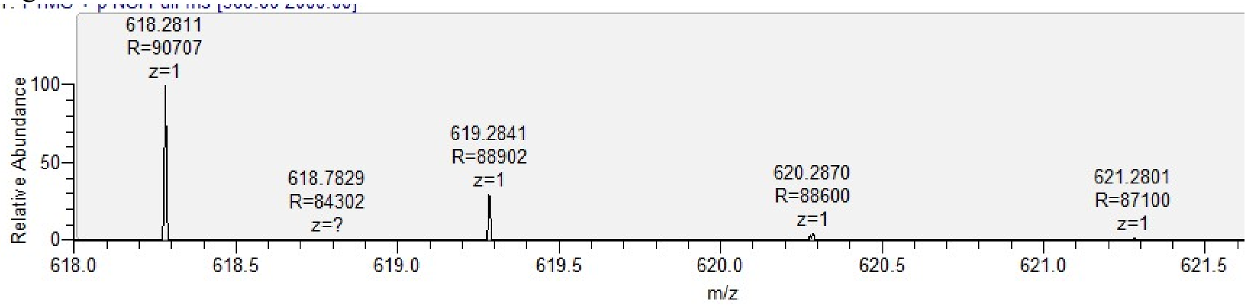

Attempted synthesis of cyclic RPTWA

Intermediate 3b: H-Arg(Pbf)-Pro-Thr(t-Bu)-Trp(Boc)-Ala-OH

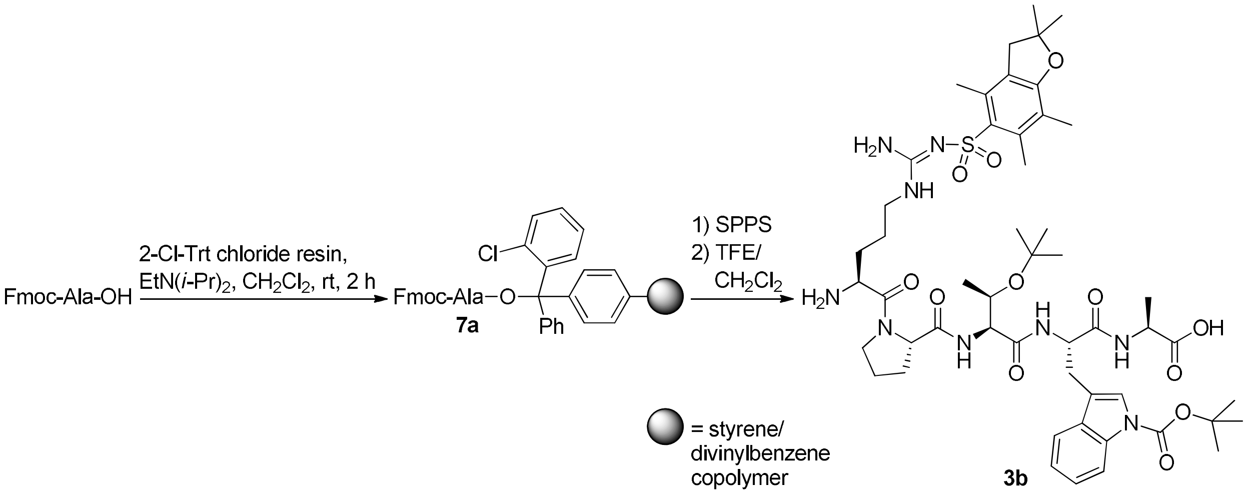

The manual Trt resin loading procedure was conducted using Fmoc-Ala-OH (93 mg, 0.3 mmol) as input to afford Fmoc-alaninyl Trt resin **7a**. Subsequent execution of the manual peptide elongation procedure using Fmoc-Trp(Boc)-OH (395 mg, 0.75 mmol), Fmoc-Thr(O*t*-Bu)-OH (298 mg, 0.750 mmol), Fmoc-Pro-OH (253 mg, 0.750 mmol), and Fmoc-Arg(Pbf)-OH (324 mg, 0.500 mmol) as inputs afforded peptidyl Trt resin free amine **2b**. Half of the latter was subjected to the TFE-mediated Trt resin cleavage procedure to ultimately afford 111.7 mg (MW 1038.26, 107.6 µmol, 86% yield) of side-chain protected peptide **3b**. HPLC-MS characterization using method “E”; retention time: 1.31 min, crude purity: 92 %, MS (ESI$^+$) *m*/*z*: $[M+H–Boc]^+$ Calcd for $C_{46}H_{68}N_9O_{10}S$ 938.5, found 938.5.

Intermediate 5b: cyclo-[(Arg(Pfb)-Pro-Thr(t-Bu)-Trp(Boc)-Ala)2]

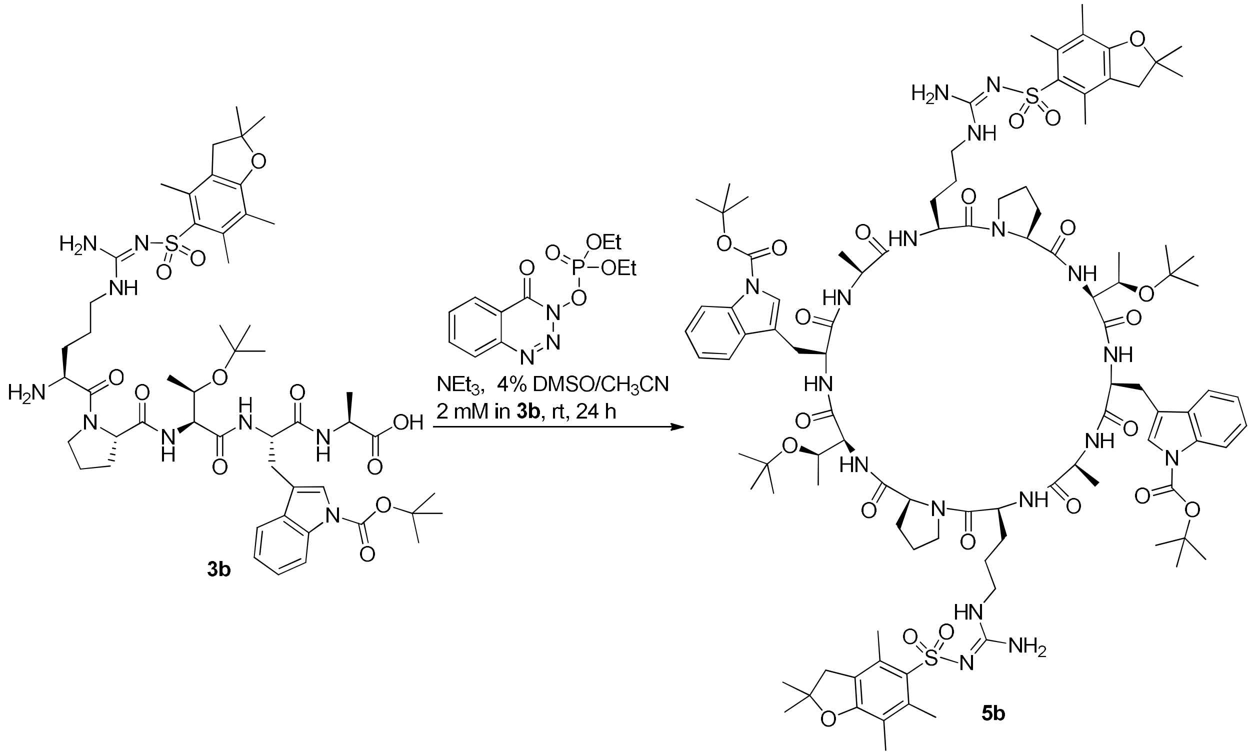

Side chain-protected peptide **3b** (20 mg, 19 µmol) was subjected to the peptide macrocyclization procedure using scaled amounts of NEt_3 (26.9 µL, 193 µmol), CH_3CN (9.7 mL), DMSO (460 µL), and DEPBT (14.4 mg, 48 µmol). Preparatory HPLC method "A" for purification ultimately afforded 3.1 mg (MW 2040.49, 1.5 µmol, 16% yield) of cyclic dimeric side product **5b**. HPLC-MS characterization using method "E"; retention time: 1.56 min, purity: >98 %, MS (ESI$^+$) *m/z*: $[M+2H]^{2+}$ Calcd for $C_{102}H_{148}N_{18}O_{22}S_2$ 1021.0, found 1021.7. HPLC-HRMS (ESI-TOF) *m/z*: $[M+2H]^{2+}$ Calcd for $C_{102}H_{148}N_{18}O_{22}S_2$ 1020.5223, found 1020.5223.

Side-product 6b: cyclo-[(Arg-Pro-Thr-Trp-Ala)$_2$]

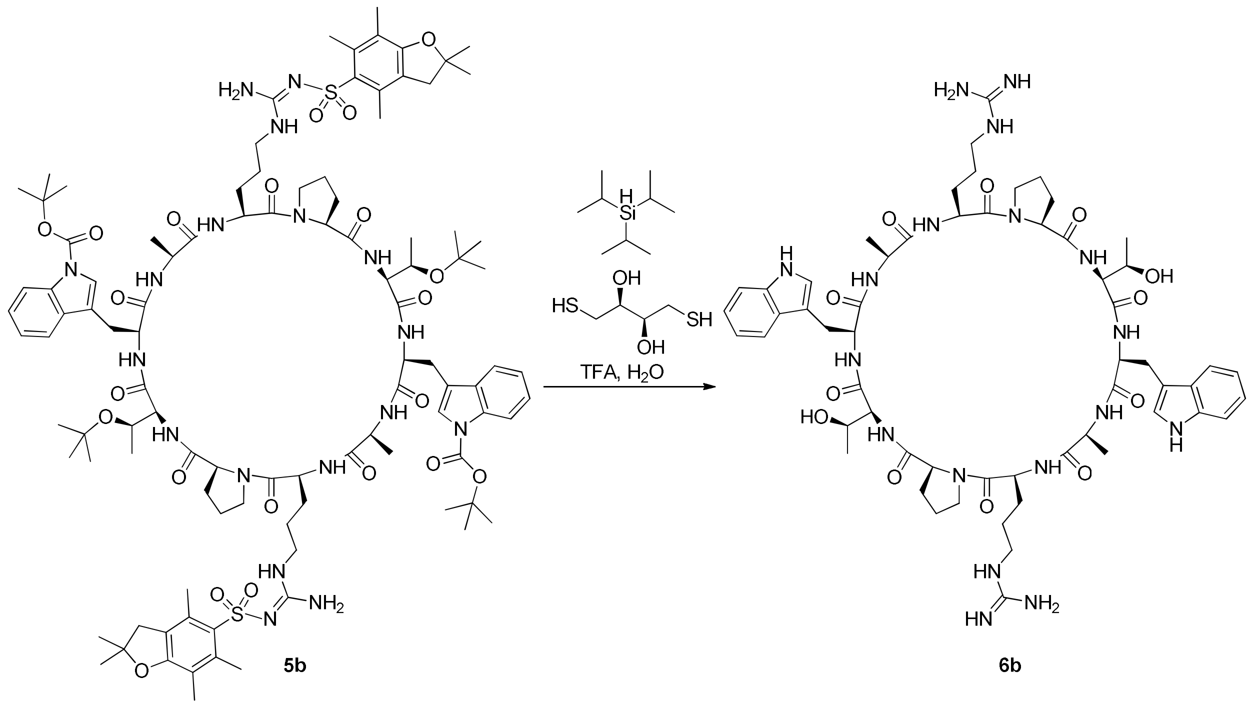

The TFA-mediated deprotection procedure was conducted on peptide **5b** (3 mg, 1.5 µmol) using scaled amounts of DTT (~0.2 mg, 1.5 µmol), H_2O (11 µl), TFA (0.20 mL), and triisopropylsilane (4.5 µl, 22 µmol). Purification was achieved by manual reversed-phase chromatography to ultimately afford 1.2 mg (bis TFA salt FW: 1451.4, 0.827 µmol, 56% yield) of *cyclo*-[argininyl-prolinyl-threoninyl-tryptophanyl-alaninyl-argininyl-prolinyl-threoninyl-tryptophanyl-alanine] (**6b**) as a solid. UHPLC-HRMS characterization using method "I"; retention time: 13.6 min, HRMS (ESI$^+$) *m/z*: $[M+H]^+$ Calcd for $C_{58}H_{83}N_{18}O_{12}$ 1223.6432, found 1223.6421; $[M+2H]^{2+}$ Calcd for $C_{58}H_{84}N_{18}O_{12}$ 612. 3253, found 612.3246. HCD MS/MS fragment count: Calcd for b/y ions 35, found 22.

Mass chromatogram of **6b** (TIC)

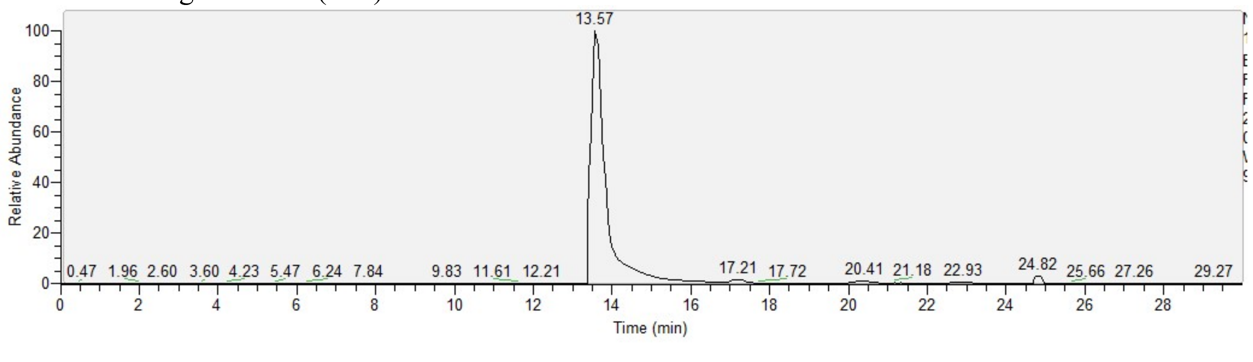

MS expansion illustrating the isotopic profile of **6b** in the singly-charged ion region

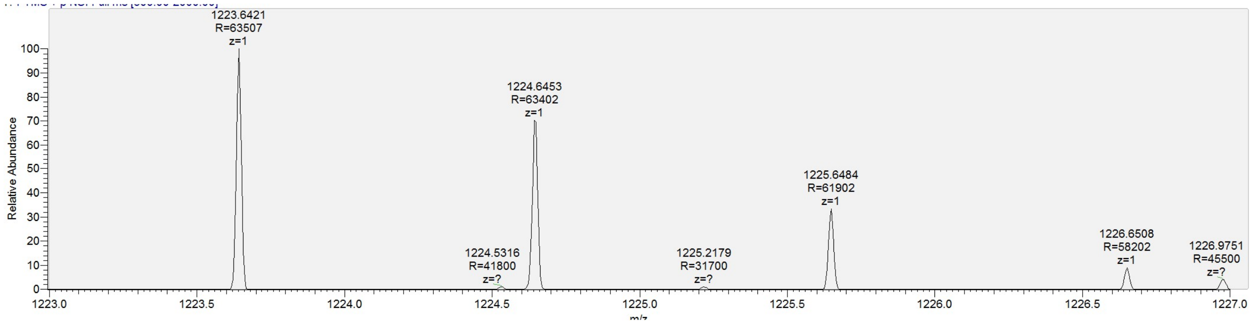

MS expansion illustrating the isotopic profile of **6b** in the doubly-charged ion region

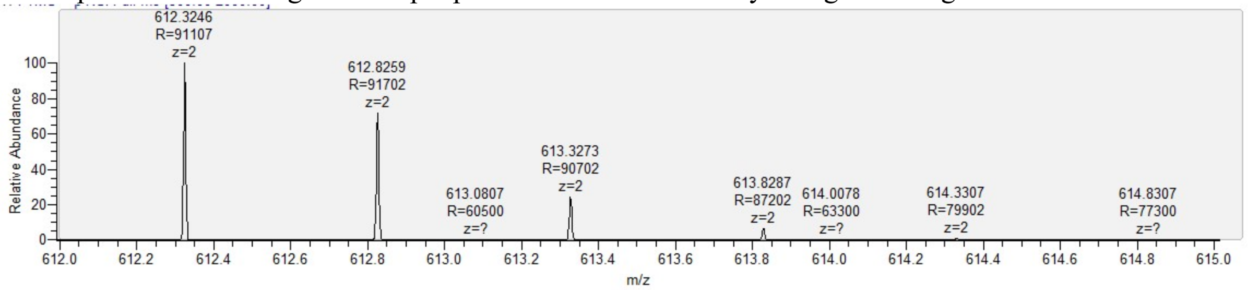

Synthesis of cyclic RATWA

Intermediate 3c: H-Arg(Pbf)-Ala-Thr(t-Bu)-Trp(Boc)-Ala-OH

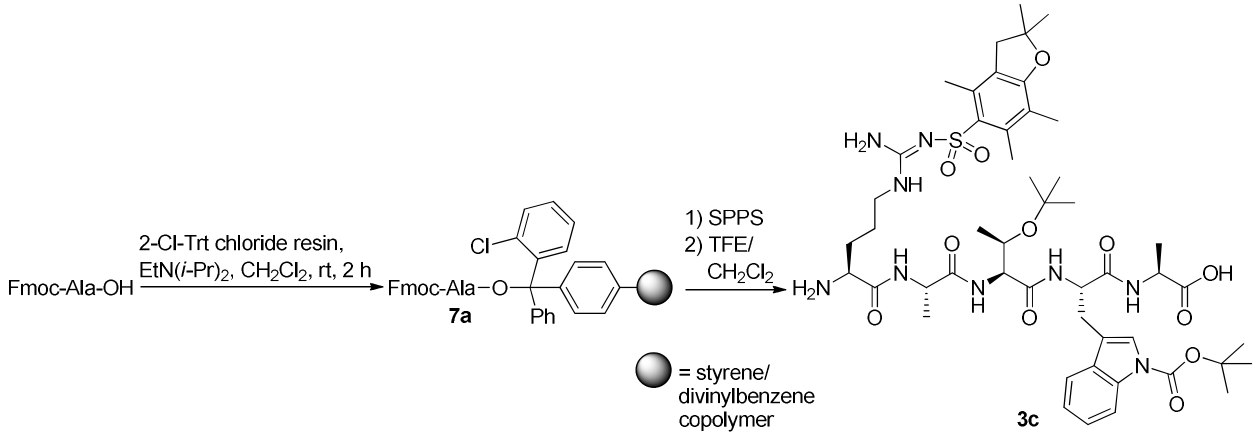

The manual Trt resin loading procedure was conducted using Fmoc-Ala-OH (93 mg, 0.3 mmol) as input to afford Fmoc-alaninyl Trt resin **7a**. Subsequent execution of the manual peptide elongation procedure using Fmoc-Trp(Boc)-OH (395 mg, 0.75 mmol), Fmoc-Thr(O*t*-Bu)-OH (298 mg, 0.750 mmol), Fmoc-Ala-OH (233 mg, 0.750 mmol), and Fmoc-Arg(Pbf)-OH (324 mg, 0.500 mmol) as inputs afforded peptidyl Trt resin free amine **2c**. Half of the latter was subjected to the TFE-mediated Trt resin cleavage procedure to ultimately afford 90 mg (MW 1012.22, 89 µmol, 71% yield) of side-chain protected peptide **3c**. HPLC-MS

characterization using method “E”; retention time: 1.28 min, crude purity: 92 %, MS (ESI$^+$) *m/z*: $[M+H]^+$ Calcd for $C_{49}H_{74}N_9O_{12}S$ 1012.5, found 1012.5.

Intermediates 4c and 5c: cyclo-[Arg(Pbf)-Ala-Thr(t-Bu)-Trp(Boc)-Ala] with dimer

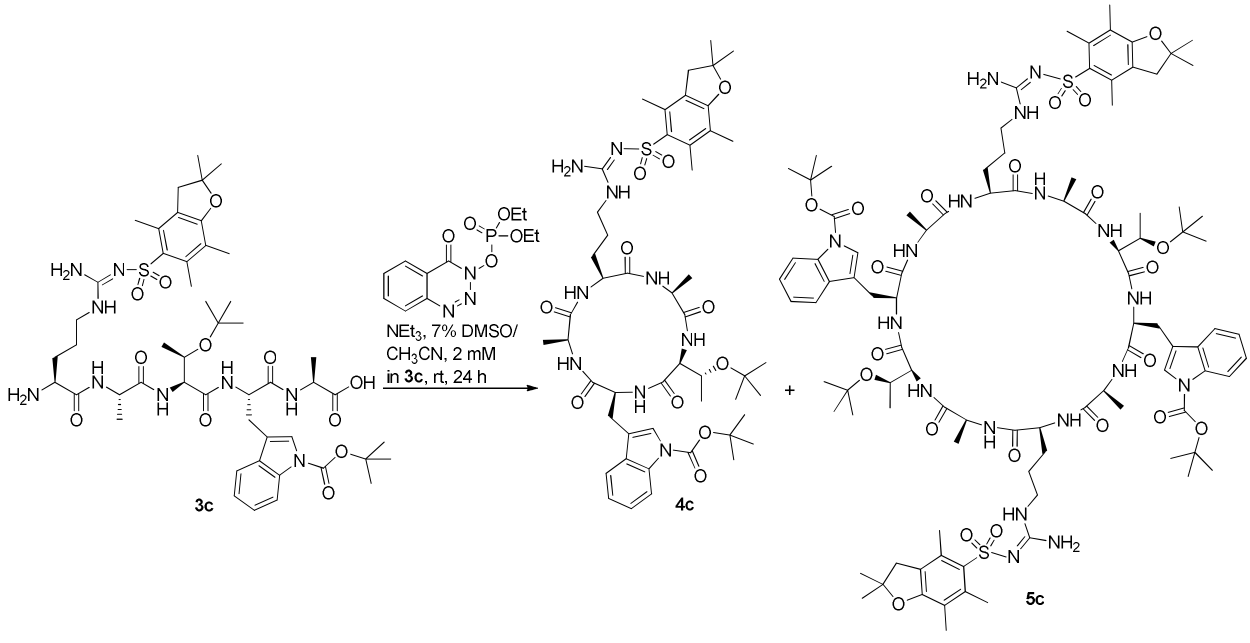

Side chain-protected peptide **3c** (26.3 mg, 26 µmol) was subjected to the peptide macrocyclization procedure using scaled amounts of NEt_3 (36 µL, 260 µmol), CH_3CN (12.5 mL), DMSO (1 mL, 7%), and DEPBT (19.4 mg, 65 µmol). Preparatory HPLC method “A” for purification ultimately afforded 7 mg (27% yield) of a mixture containing cylic peptide **4c** and dimeric side product **5c**. HPLC-MS characterization using method “E”; retention time: 1.54 min, purity: >99 %, MS (ESI$^+$) *m/z*: [**4c**+H]$^+$ Calcd for $C_{49}H_{72}N_9O_{11}S$ 994.5, found 994.5; and [**5c**+H]$^+$ Calcd for $C_{97}{}^{13}CH_{143}N_{18}O_{22}S_2$ 1990.0, found 1990.1; Calcd for $C_{98}H_{143}N_{18}O_{22}S_2$ 1989.0 found 1988.8. HRMS (ESI-TOF) *m/z*: [**5c**+H]$^+$ Calcd for $C_{98}H_{143}N_{18}O_{22}S_2$ 1988.0061, found 1988.0060; [**5c**+Na]$^+$ Calcd for $C_{98}H_{143}N_{18}NaO_{22}S_2$ 2009.9880, found 2009.9880. The **4c**/**5c** ratio was ascertained from analysis of deptrotected derivatives **1c**/**6c**. Note that the high molecular weight of **5c** causes the abundance of the ^{13}C isotope to surpass its natural isotope.

Product 1c/ side-product 6c mixture: cyclo-[Arg-Ala-Thr-Trp-Ala] with dimer

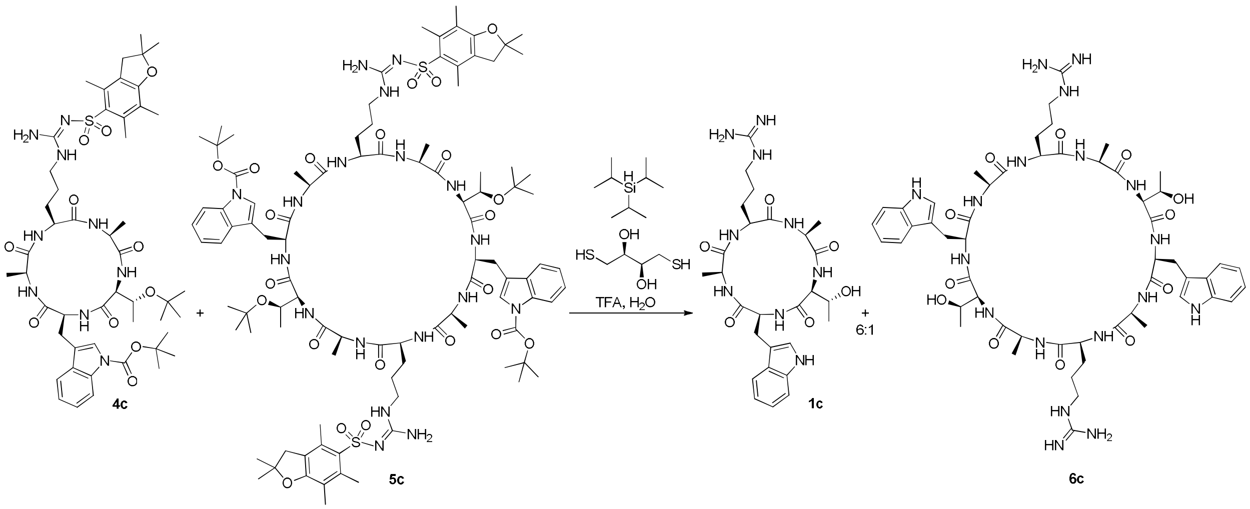

The TFA-mediated deprotection procedure was conducted on the mixture containing cylic peptide **4c** and dimeric side product **5c** (7 mg, 7 µmol) using scaled amounts of DTT (1 mg, 7 µmol), H_2O (53 µl), TFA (0.96 mL), and triisopropylsilane (21.6 µl, 105 µmol). The reaction time was adjusted to 1 h, and purification was achieved by manual reversed-phase chromatography to ultimately afford 3.2 mg (major component TFA salt FW: 699.7, 3 µmol, 65%) of a solid mixture containing *cyclo*-[argininyl-alaninyl-threoninyl-tryptophanyl-alanine] (**1c**) and *cyclo*-[argininyl-alaninyl-threoninyl-tryptophanyl-alaninyl-argininyl-alaninyl-threoninyl-tryptophanyl-alanine] (**6c**) in a ratio of 86:14. UHPLC-HRMS characterization using method "I"; retention time: 13.8 min, HRMS (ESI^+) *m/z*: [**1c**+H]$^+$ Calcd for $C_{27}H_{40}N_9O_6$ 586.3096, found 586.3164; [**6c**+H]$^+$ Calcd for $C_{54}H_{79}N_{18}O_{12}$ 1171.6119, found 1171.6094. HCD MS/MS fragment count (**1c**): Calcd for b/y ions 35, found 30.

Mass chromatogram of the **1c**/**6c** mixture (TIC)

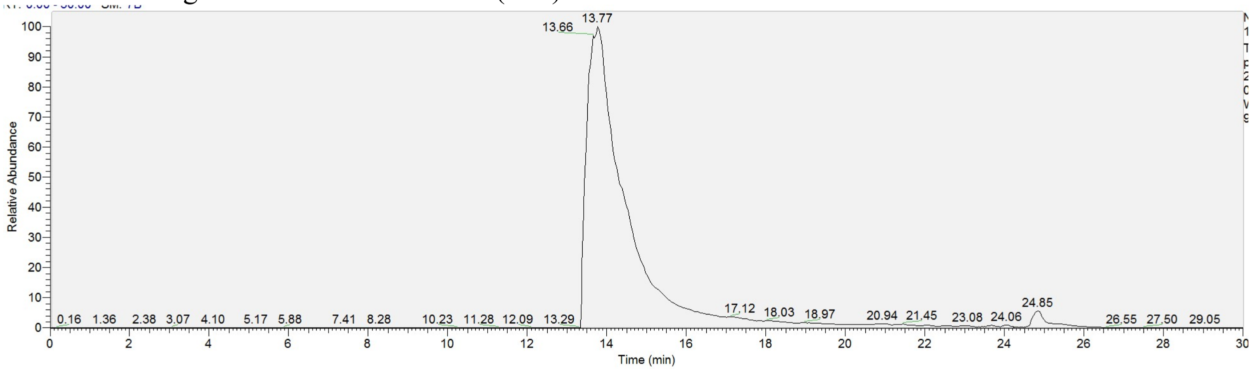

Mass chromatogram after extracting **1c**-ions

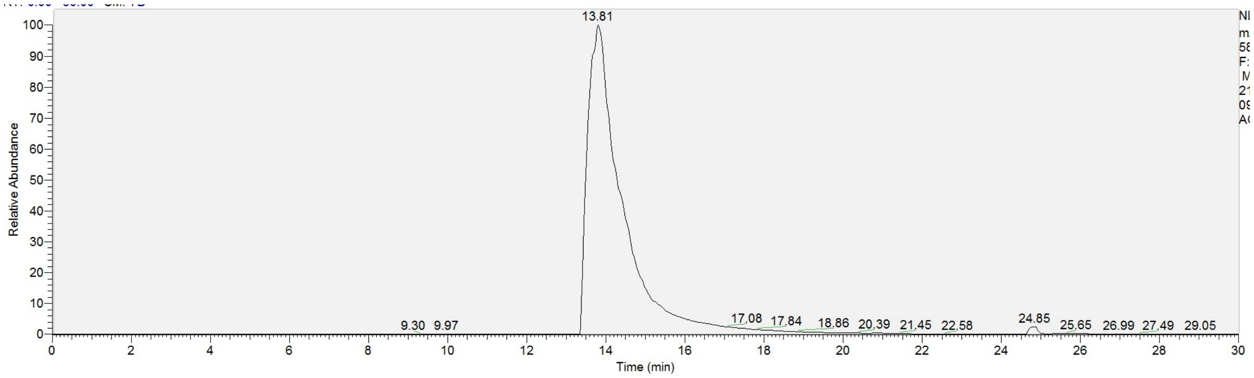

MS expansion illustrating the isotopic profile of **1c**

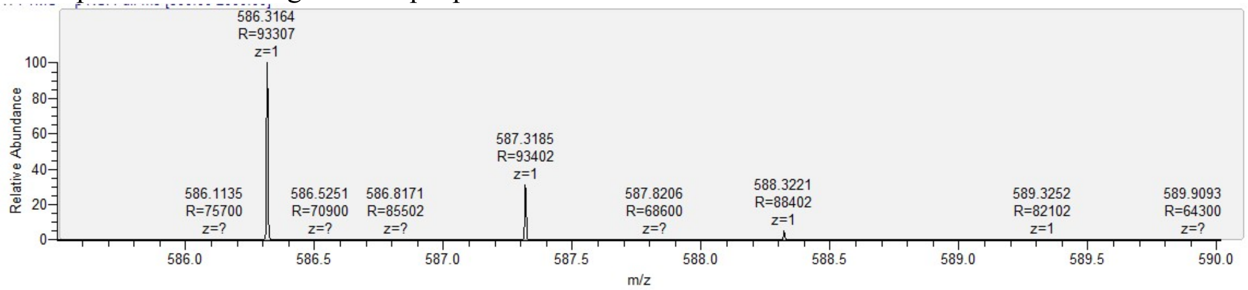

Mass chromatogram after extracting **6c**-ions

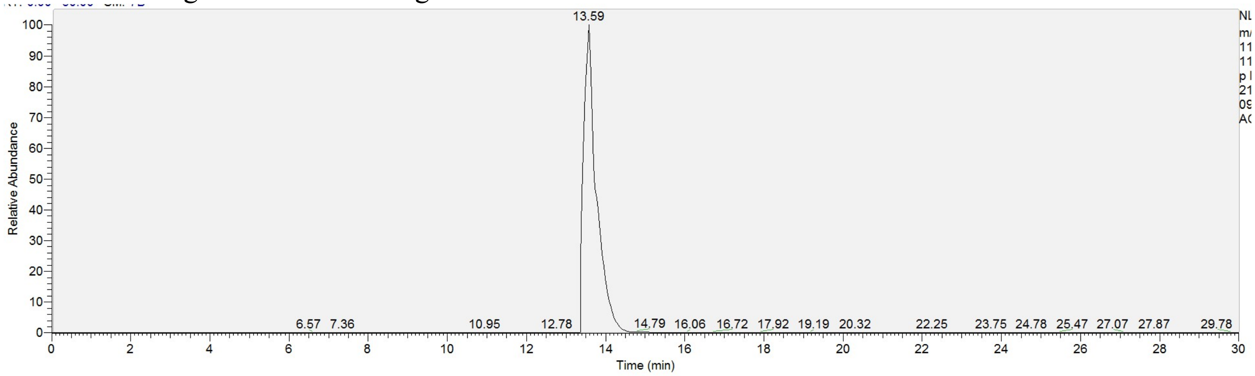

MS expansion illustrating the isotopic profile of **6c**

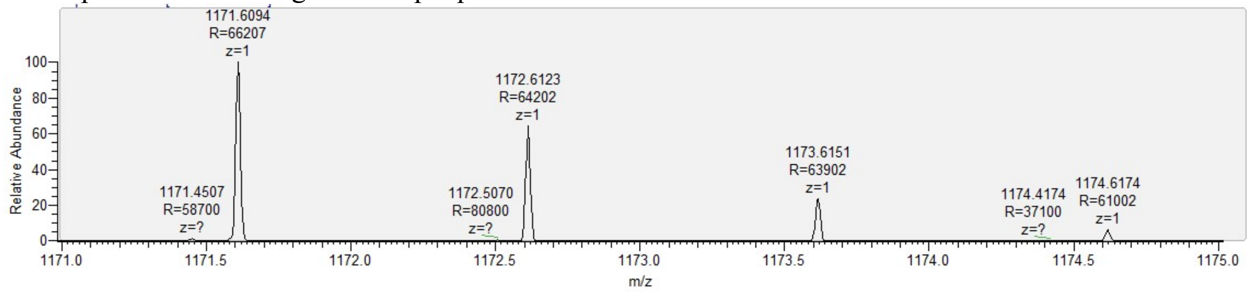

Synthesis of cyclic RCTCA

Intermediate 3d: H-Arg(Pbf)-Pro-Thr(t-Bu)-Cys(Trt)-Ala-OH

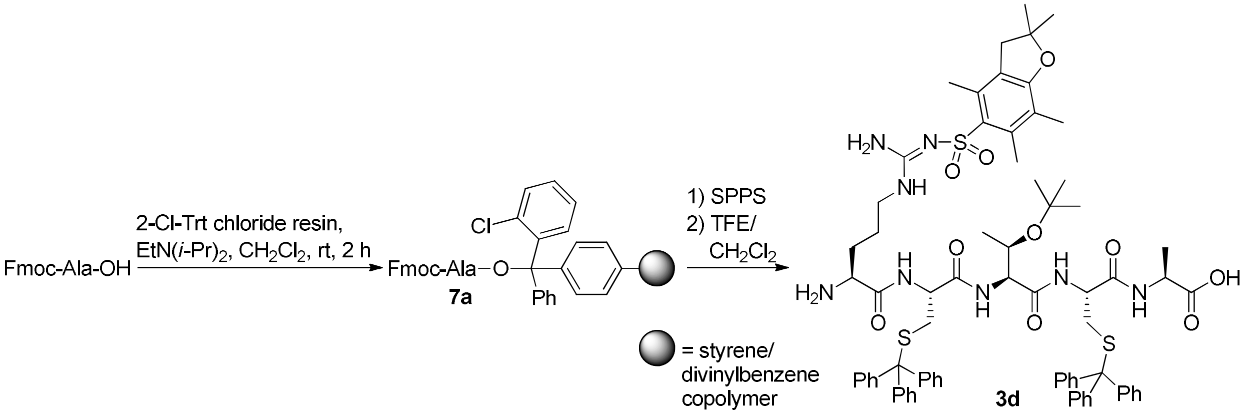

The manual Trt resin loading procedure was conducted using Fmoc-Ala-OH (93 mg, 0.3 mmol) as input to afford Fmoc-alaninyl Trt resin **7a**. Subsequent execution of the manual peptide elongation procedure using Fmoc-Cys(Trt)-OH (293 mg, 0.500 mmol), Fmoc-Thr(O*t*-Bu)-OH (298 mg, 0.750 mmol), Fmoc-Cys(Trt)-OH (293 mg, 0.500 mmol), and Fmoc-Arg(Pbf)-OH (324 mg, 0.500 mmol) as inputs afforded peptidyl Trt resin free amine **2d**. Half of the latter was subjected to the TFE-mediated Trt resin cleavage procedure to afford 144 mg (MW 1345.73, 107 µmol, 86% yield) of side-chain protected peptide **3d**. HPLC-MS characterization using method "E"; retention time: 1.98 min, crude purity: 92 %, MS (ESI$^+$) *m*/*z*: $[M+H]^+$ Calcd for $C_{74}H_{89}N_8O_8S_3$ 1345.6, found 1346.0.

Intermediate 4d and 5d: cyclo-[Arg(Pbf)-Cys(Trt)-Thr(t-Bu)-Cys(Trt)-Ala] with dimer

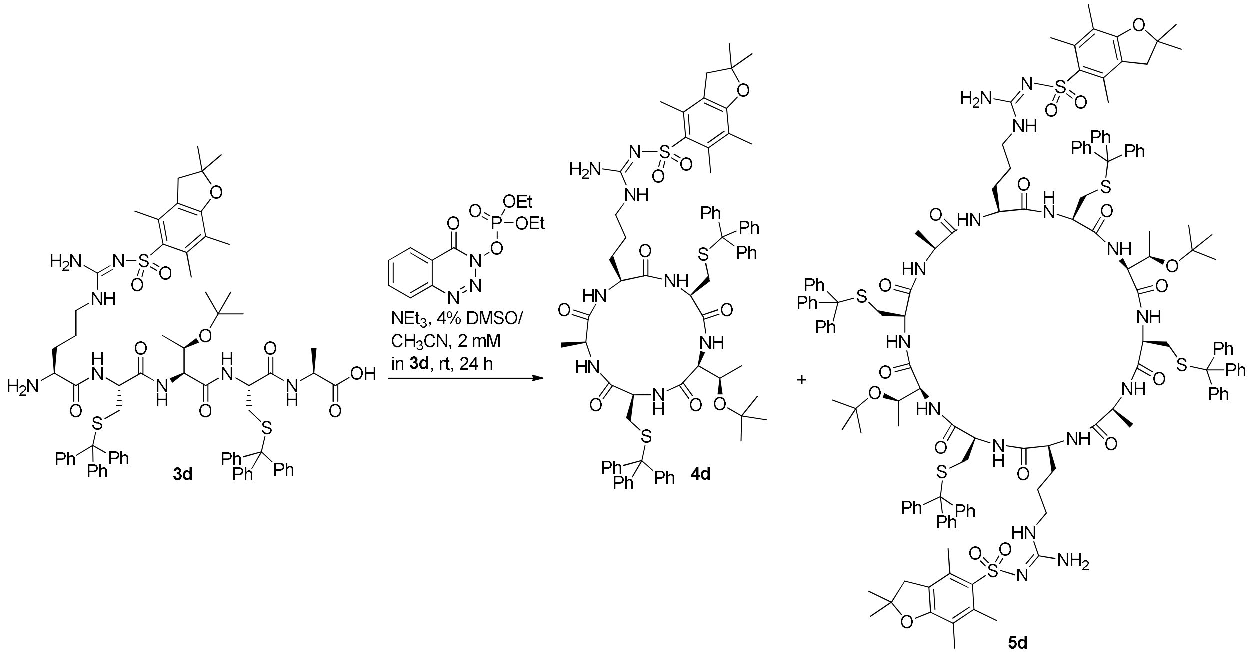

Side chain-protected peptide **3d** (21.5 mg, 16 µmol) was subjected to the peptide macrocyclization procedure using scaled amounts of NEt_3 (22.3 µL, 260 µmol), CH_3CN (7.7 mL), DMSO (320 µL), and DEPBT (12 mg, 40 µmol). Preparatory HPLC method "A" for purification ultimately afforded 3.3 mg (16% yield) of a mixture containing cylic peptide **4d** and dimeric side product **5d**. HPLC-MS characterization using method "E"; retention time: 1.80 min, MS (ESI$^+$) *m/z*: [**4d**+H]$^+$ Calcd for $C_{74}H_{87}N_8O_8S_3$ 1327.6, found 1327.2. HRMS (ESI-TOF) *m/z*: [**4d**+H]$^+$ Calcd for $C_{74}H_{87}N_8O_8S_3$ 1327.5753, found 1327.5718. The **4d**/**5d** ratio was ascertained from analysis of deprotected derivatives **1d**/**7d**.

Product 1d/ side-product 6d mixture: cyclo-[Arg-Cys-Thr-Cys-Ala] with dimer

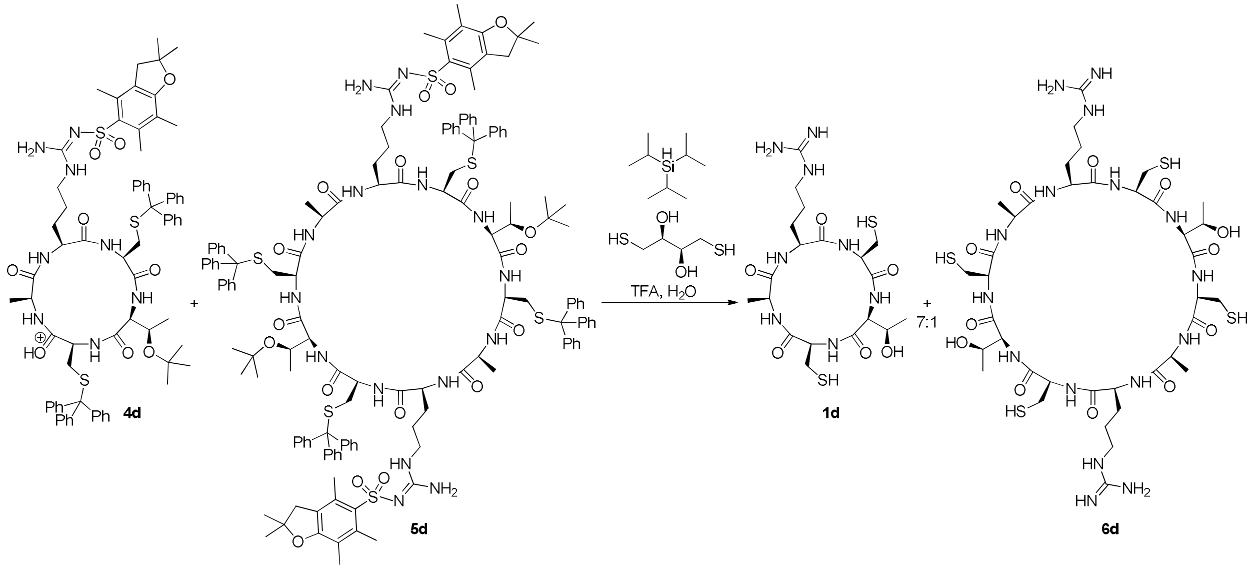

The TFA-mediated deprotection procedure was conducted on the mixture containing cylic peptide **4d** and dimeric side product **5d** (3 mg, 2.3 µmol) using scaled amounts of DTT (~0.3 mg, 2.3 µmol), H_2O (17 µl), TFA (0.31 mL), and triisopropylsilane (6.9 µl, 34 µmol). Purification was achieved by manual reversed-phase chromatography to afford 0.7 mg (major component TFA salt FW: 648.68, 1.1 µmol, 48% yield) of a solid mixture containing *cyclo*-[argininyl-cysteinyl-threoninyl-cysteinyl-alanine] (**1d**) and *cyclo*-[argininyl-cysteinyl-threoninyl-cysteinyl-alaninyl-argininyl-cysteinyl-threoninyl-cysteinyl-alanine] (**6d**) in a ratio of 88:12. UHPLC-HRMS characterization using method "I"; retention time: 13.6 min, HRMS (ESI^+) *m*/*z*: $[\mathbf{1d}+H]^+$ Calcd for $C_{19}H_{35}N_8O_6S_2$ 535.2115, found 535.2116; $[\mathbf{6d}+H]^+$ Calcd for $C_{38}H_{69}N_{16}O_{12}S_4$ 1069.4158, found 1069.4125. HCD MS/MS fragment count (**1d**): Calcd for b/y ions 35, found 32.

Mass chromatogram of the **1d**/**6d** mixture (TIC)

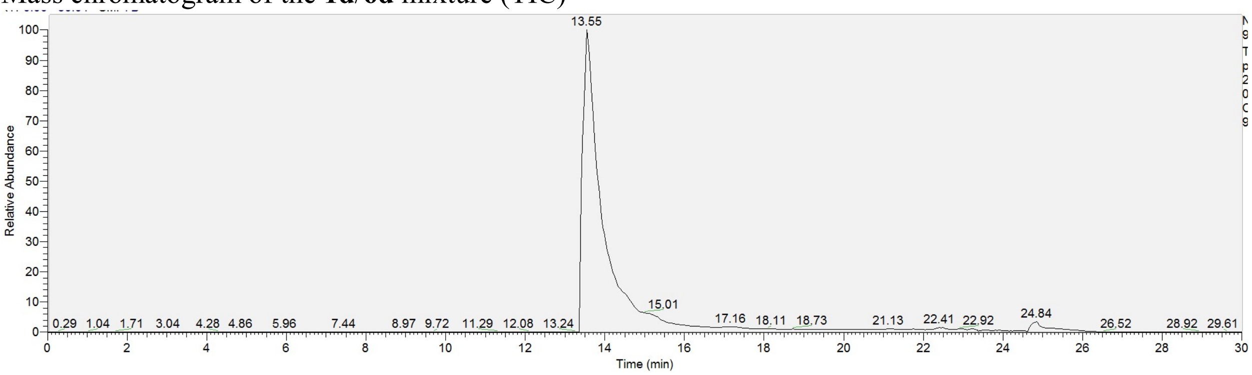

Mass chromatogram after extracting **1d**-ions

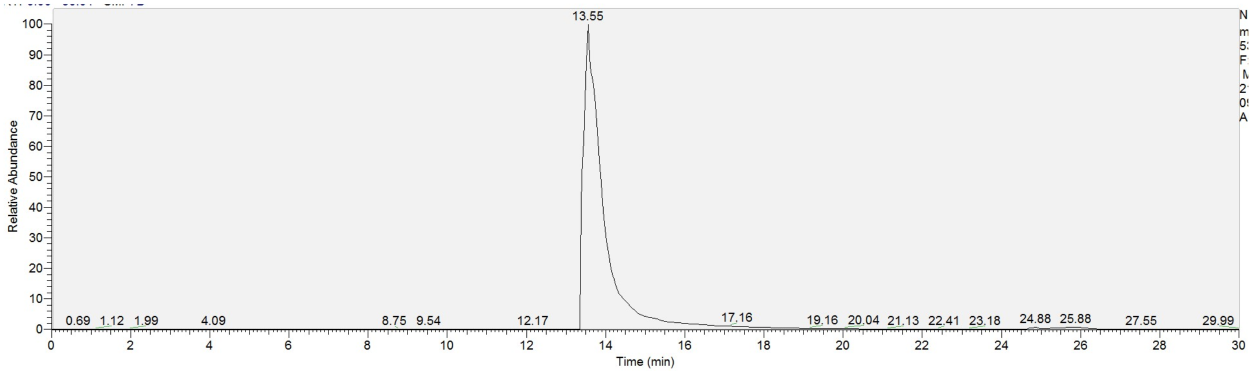

MS expansion illustrating the isotopic profile of **1d**

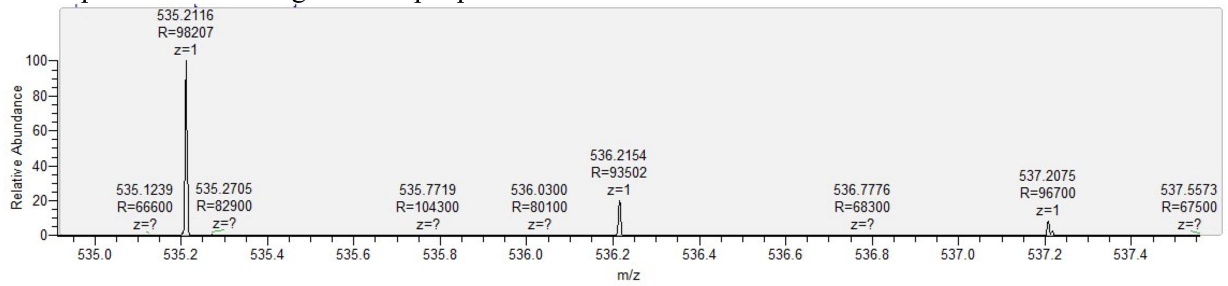

Mass chromatogram after extracting **6d**-ions

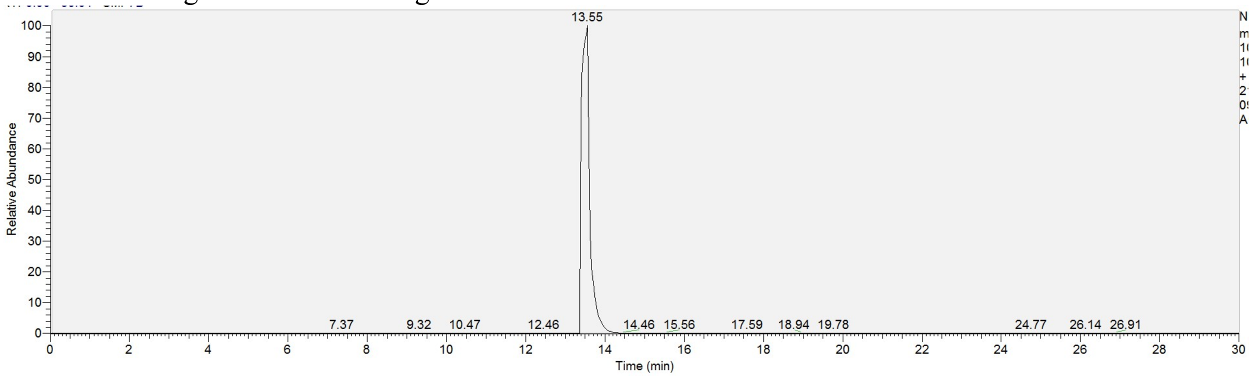

MS expansion illustrating the isotopic profile of **6d**

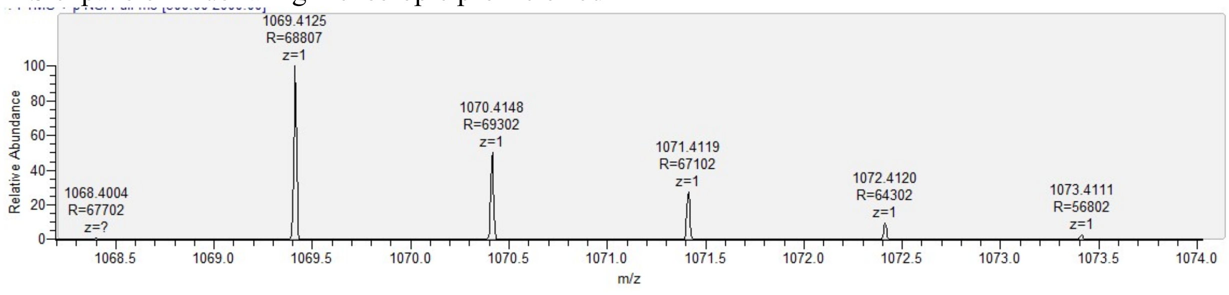

Synthesis of cyclic RCTAA

Intermediate 3e: H-Arg(Pbf)-Cys(Trt)-Thr(t-Bu)-Ala-Ala-OH

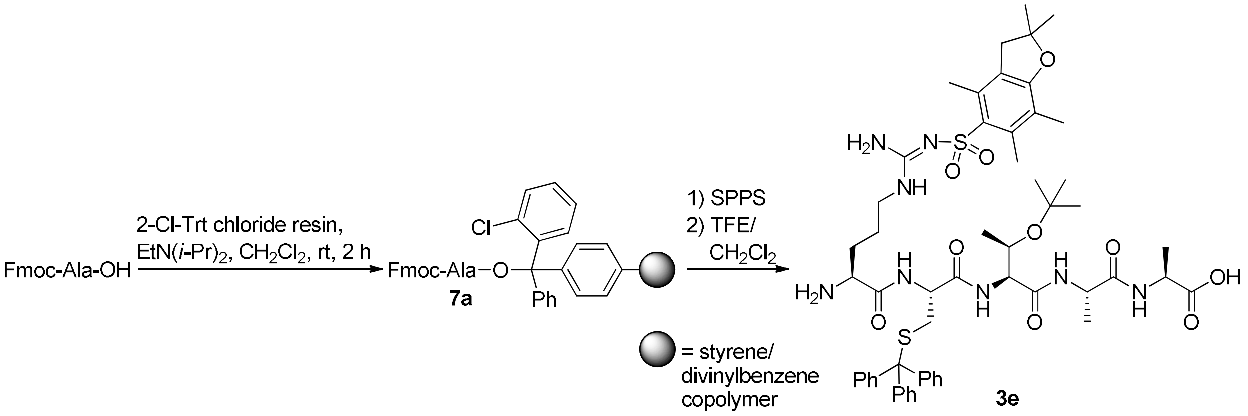

The manual Trt resin loading procedure was conducted using Fmoc-Ala-OH (93 mg, 0.3 mmol) as input to afford Fmoc-alaninyl Trt resin **7a**. Subsequent execution of the manual peptide elongation procedure using Fmoc-Ala-OH (233 mg, 0.750 mmol), Fmoc-Thr(O*t*-Bu)-OH (298 mg, 0.750 mmol), Fmoc-Cys(Trt)-OH (293 mg, 0.500 mmol), and Fmoc-Arg(Pbf)-OH (324 mg, 0.500 mmol) as inputs affordeded peptidyl Trt resin free amine **2e**. Half of the latter was subjected to the TFE-mediated Trt resin cleavage procedure to afford 126.6 mg (MW 1071.35, 118 µmol, 95% yield) of side-chain protected peptide **3e**. HPLC-MS characterization using method "E"; retention time: 1.58 min, crude purity: 72 %, MS (ESI$^+$) *m*/*z*: $[M+H]^+$ Calcd for $C_{55}H_{75}N_8O_{10}S_2$ 1071.5, found 1071.2.

Intermediate 4e: cyclo-[Arg(Pbf)-Cys(Trt)-Thr(t-Bu)-Ala-Ala]

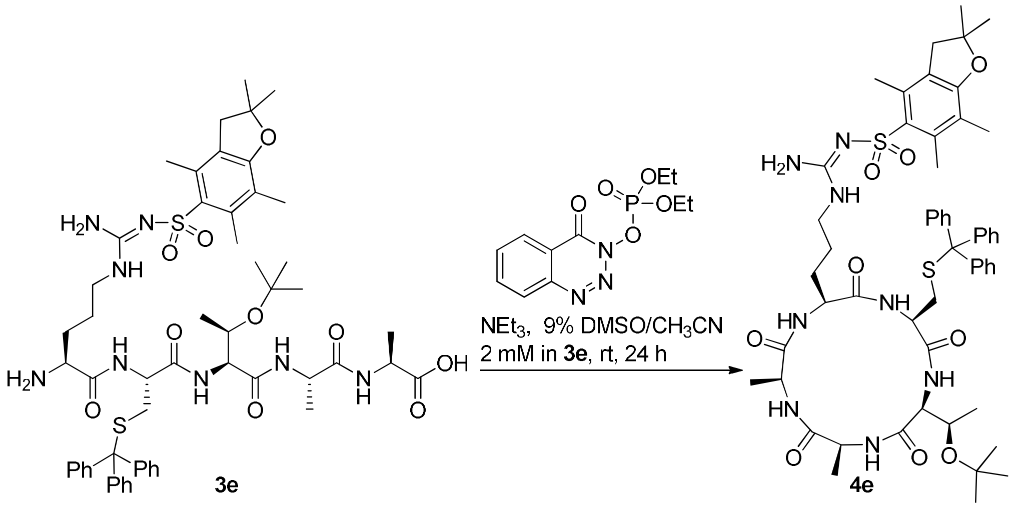

Side chain-protected peptide **3e** (22 mg, 21 µmol) was subjected to the peptide macrocyclization procedure using scaled amounts of NEt_3 (28.6 µL, 205 µmol), CH_3CN (9.3 mL), DMSO (933 µL), and DEPBT (15.4 mg, 51 µmol). Preparatory HPLC method "A" for purification ultimately afforded 5.1 mg (21% yield) protected cyclic peptide **4a**. HPLC-MS characterization using method "F"; retention time: 1.60 min, purity: >99 %, MS (ESI$^+$) *m/z*: $[M+H]^+$ Calcd for $C_{55}H_{73}N_8O_9S_2$ 1053.5, found 1054.4. HRMS (ESI-TOF) *m/z*: [**4e**+H]$^+$ Calcd for $C_{55}H_{73}N_8O_9S_2$ 1053.4936, found 1054.4952; [**4e**+Na]$^+$ Calcd for $C_{55}H_{72}N_8NaO_9S_2$ 1057.4756, found 1075.4755; a trace of dimeric byproduct **5e** was also identified: [**5e**+H]$^+$ Calcd for $C_{110}H_{145}N_{16}O_{18}S_4$ 2105.9834, found 2105.9866.

Product 1e: cyclo-[Arg-Cys-Thr-Ala-Ala]

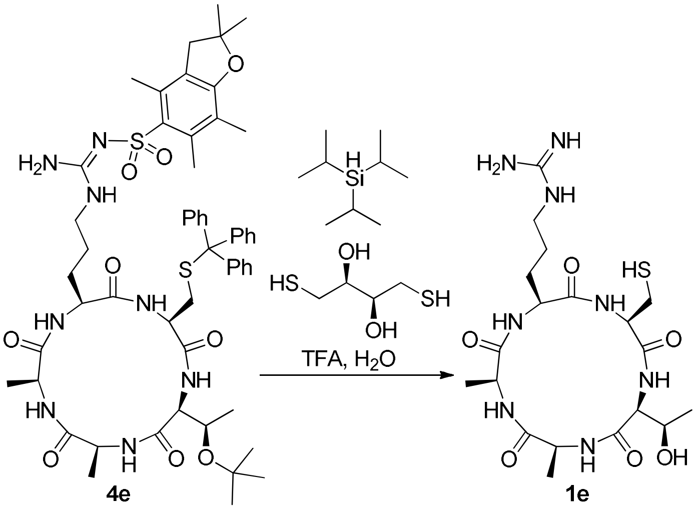

4e **1e**

The TFA-mediated deprotection procedure was conducted on peptide **4e** (4.5 mg, 4.3 µmol) using scaled amounts of DTT (~0.6 mg, 4.3 µmol), H_2O (33 µl), TFA (0.58 mL), and triisopropylsilane (13.1 µl, 64 µmol). Purification was achieved by manual reversed-phase chromatography to afford 1.9 mg (TFA salt FW: 616.6, 3.1 µmol, 72% yield) of *cyclo*-[argininyl-cysteinyl-threoninyl-alaninyl-alanine] (**1e**) as a solid. UHPLC-HRMS characterization using method "I"; retention time: 13.6 min, HRMS (ESI$^+$) *m/z*: $[M+H]^+$ Calcd for $C_{19}H_{35}N_8O_6S$ 503.2395, found 503.2390. HCD MS/MS fragment count: Calcd for b/y ions 35, found 32.

Mass chromatogram of **1e** (TIC)

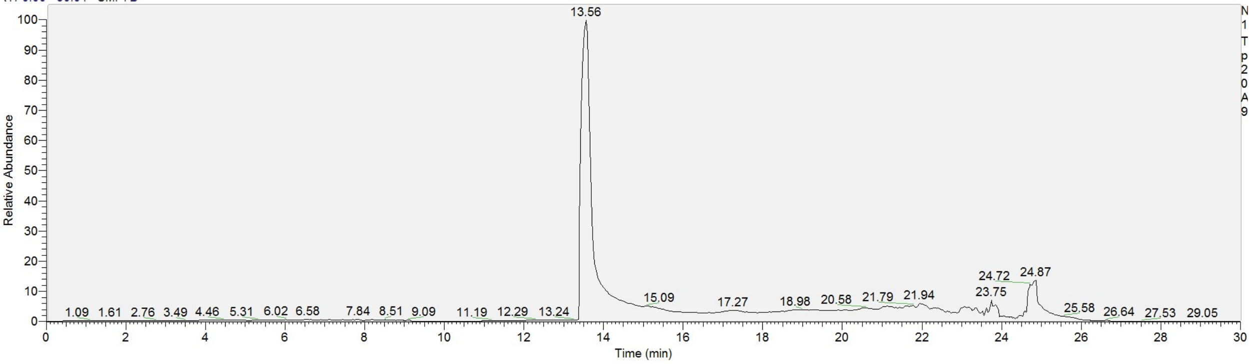

MS expansion illustrating the isotopic profile of **1e**

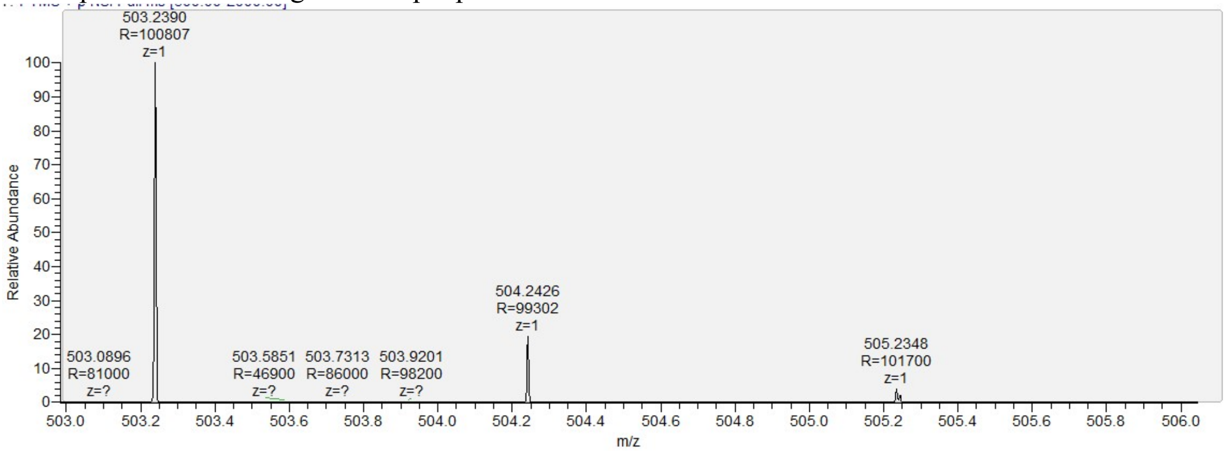

Synthesis of cyclic WCKPIPT

Intermediate 3f: H-Trp(Boc)-Cys(Trt)-Lys(Boc)-Pro-Ile-Pro-Thr(O-tBu)-OH

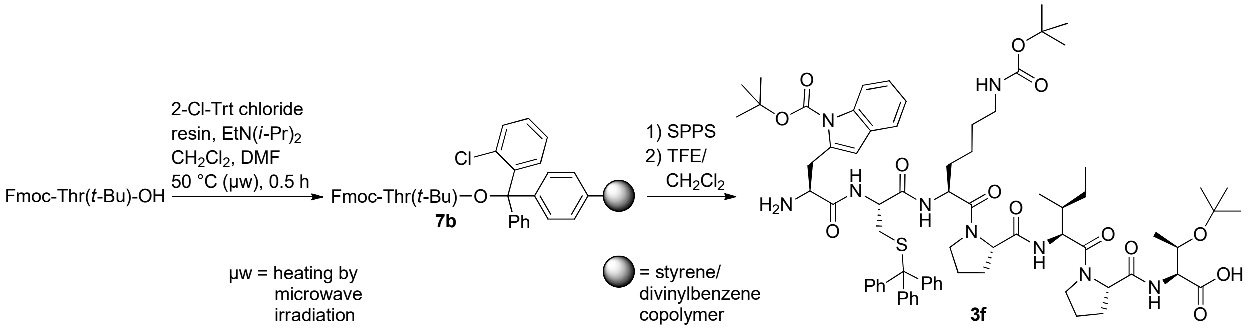

The automated Trt resin loading procedure was executed using a CH_2Cl_2 solution of Fmoc-Thr(*t*-Bu)-OH (5 mL, 0.2 M, 1 mmol) to afford Fmoc-threoninyl Trt resin **7b**. Continuation of SPPS by the automated peptide elongation procedure using standard DMF solutions (5 mL, 0.2 M, 4 equiv.) of Fmoc-Pro-OH, Fmoc-Ile-OH, Fmoc-Pro-OH, Fmoc-Lys(Boc)-OH, Fmoc-Cys(Trt)-OH, and Fmoc-Trp(Boc)-OH as input

ultimately afforded *N*-terminal free amine peptidyl resin **2f**. The latter was subjected to the TFE-mediated Trt resin cleavage procedure using adjusted solvent (15 mL) and time (2x 1 h) quantities to afford 166 mg (FW 1342.68, 125 µmol, 50% yield) of side chain-protected peptide **3f**. HPLC-MS characterization using method "G"; retention time: 2.36 min, crude purity: 95 %, MS (ESI$^+$) *m*/*z*: $[M+H]^+$ Calcd for $C_{73}H_{100}N_9O_{13}S$ 1342.7, found 1342.7.

Intermediate 4f: cyclo-[Trp(Boc)-Cys(Trt)-Lys(Boc)-Pro-Ile-Pro-Thr(O-tBu)]

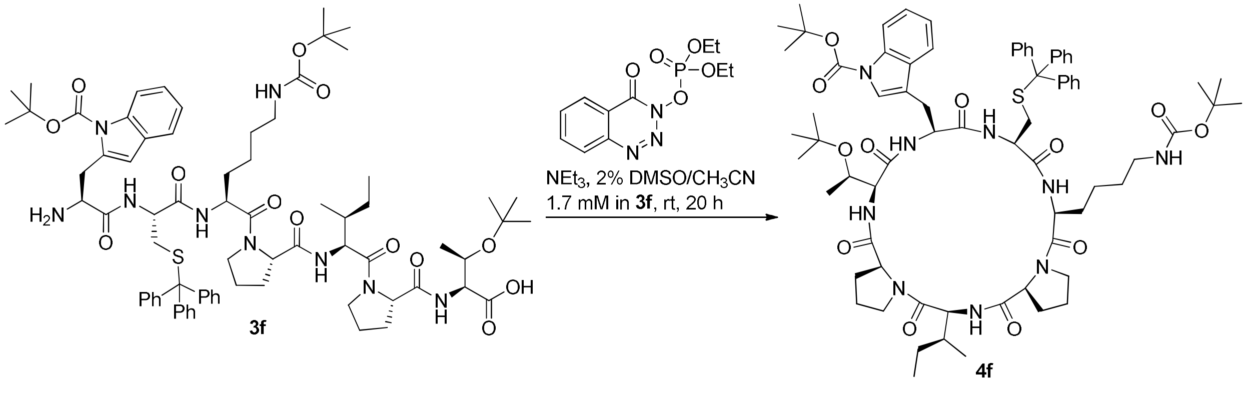

Side chain-protected peptide **3f** (143 mg, 107 µmol) was subjected to the peptide macrocyclization procedure using scaled amounts of NEt_3 (178 µL, 1.28 mmol), CH_3CN (60 mL), DMSO (1-1.5 mL), and DEPBT (80 mg, 266 µmol). Preparatory HPLC method "C" for purification, collecting the peak that eluted at 9.80 min, ultimately afforded 17.5 mg (MW 1324.67, 13.0 µmol, 12% yield) of protected cyclic peptide **4f**. HPLC-MS characterization using method "G"; retention time: 3.41 min, purity: 98 %., MS (ESI$^+$) *m*/*z*: $[M+H–Boc]^+$ Calcd for $C_{68}H_{90}N_9O_{10}S$ 1224.7, found 1224.6.

Product 1f: cyclo-[Trp-Cys-Lys-Pro-Ile-Pro-Thr]

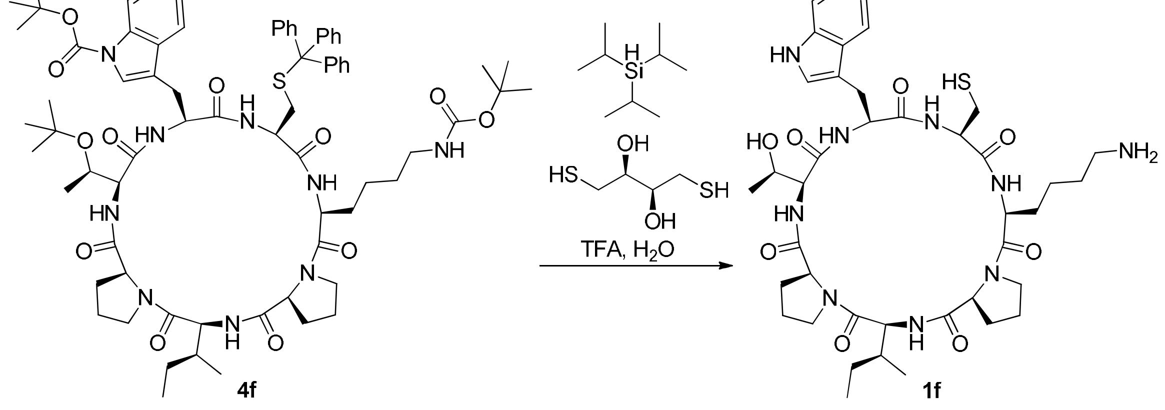

The TFA-mediated deprotection procedure was conducted on peptide **4f** (17.5 mg, 13 µmol) using scaled amounts of DTT (~1 mg, ~6.5 µmol), H_2O (55 µl), TFA (0.99 mL), and triisopropylsilane (54.1 µl, 264 µmol). Purification was achieved by preparative HPLC-MS using method "C" to ultimately afford 4.4 mg (HCl salt FW: 862.5, 3 µmol, 39% yield) of *cyclo*-[tryptophanyl-cysteinyl-lysinyl-prolinyl-isoleucinyl-prolinyl-threonine] (**1f**) as a solid. UHPLC-HRMS characterization using method "I"; retention time: 13.6 min, HRMS (ESI$^+$) *m/z*: $[M+H]^+$ Calcd for $C_{40}H_{60}N_9O_8S$ 826.4280, found 826.4285. HCD MS/MS fragment count: Calcd for b/y ions 77, found 56. Peak tailing in the UHPLC trace was attributed to the presence of slowly equilibrating conformational isomers.

Mass chromatogram of **1f** (TIC)

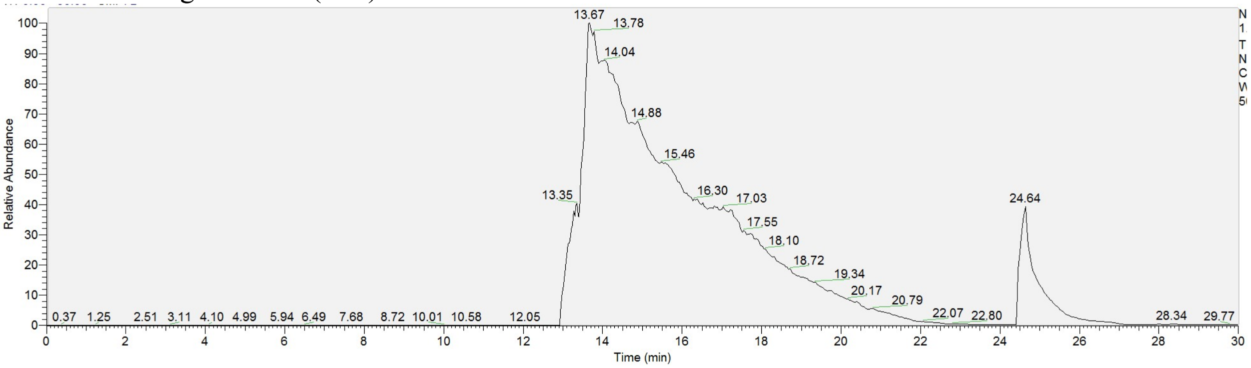

MS expansion illustrating the isotopic profile of **1f**

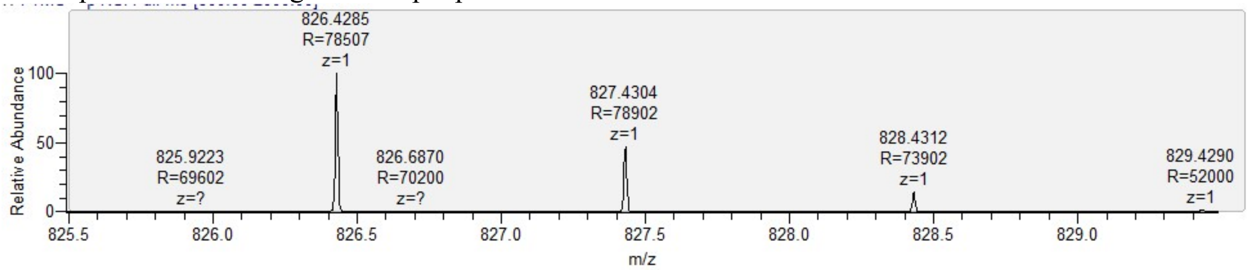

Higher-energy Collisional Dissociation Tandem Mass Spectrometry (HCD MS/MS) fragmentation spectrum of **1f** for sequence confirmation (For clarity, annotations for selected *b* and *y* peptide fragment ions are shown. Standard peptidic fragment ion nomenclature[7] was adapted to cyclic peptide **1f** using the illustrated arbitrary 1–7 numerical assignments for the isomeric ring-opening intermediates).

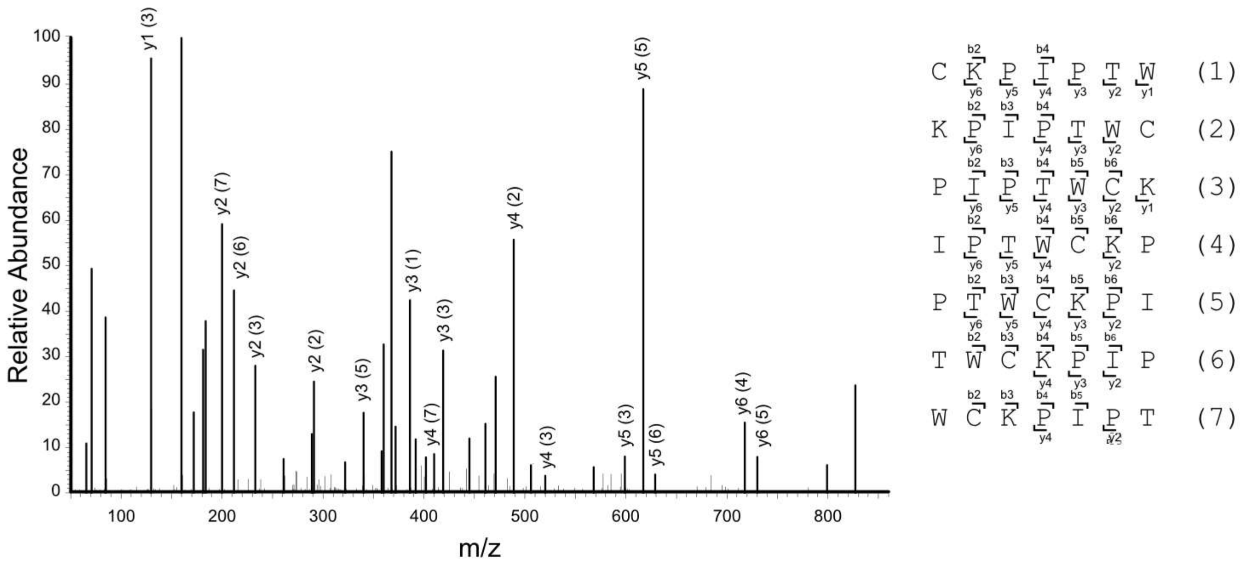

Synthesis of cyclic KCKPFKSI

Intermediate 3g: H-Lys(Boc)-Cys(Trt)-Lys(Boc)-Pro-Phe-Lys(Boc)-Ser(t-Bu)-Ile-OH

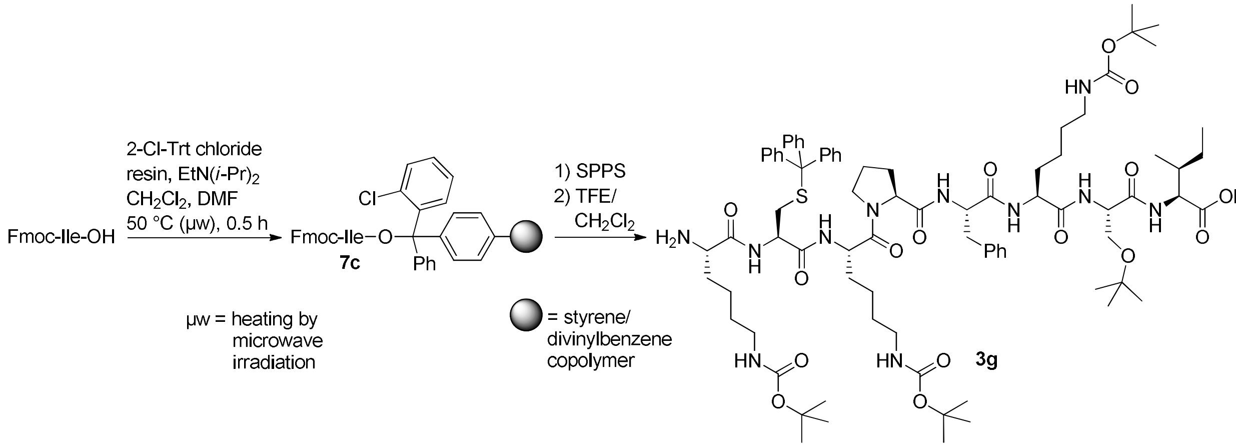

The automated Trt resin loading procedure was executed using a CH_2Cl_2 solution of Fmoc-Ile-OH (5 mL, 0.2 M, 1 mmol) to afford Fmoc-isoleucinyl Trt resin **7c**. Continuation of SPPS by the automated peptide elongation procedure using standard DMF solutions (5 mL, 0.2 M, 4 equiv.) of Fmoc-Ser(*t*-Bu)-OH, Fmoc-Lys(Boc)-OH, Fmoc-Phe-OH, Fmoc-Pro-OH, Fmoc-Lys(Boc)-OH, Fmoc-Cys(Trt)-OH, and Fmoc-Lys(Boc)-OH as inputs afforded *N*-terminal free amine peptidyl resin **2g**. The latter was subjected to the TFE-mediated Trt resin cleavage procedure using an adjusted volume of TFE/ CH_2Cl_2 solvent (20 mL) to ultimately afford 170 mg (MW 1548.97, 110 μmol, 44% yield) of side chain-protected peptide **3g**. HPLC-

MS characterization using method “G”; retention time: 2.33 min, crude purity: 94 %, MS (ESI$^+$) *m/z*: $[M+H]^+$ Calcd for $C_{82}H_{122}N_{11}O_{16}S$ 1548.9, found 1548.7.

Intermediate 4g: cyclo-[Lys(Boc)-Cys(Trt)-Lys(Boc)-Pro-Phe-Lys(Boc)-Ser(t-Bu)-Ile]

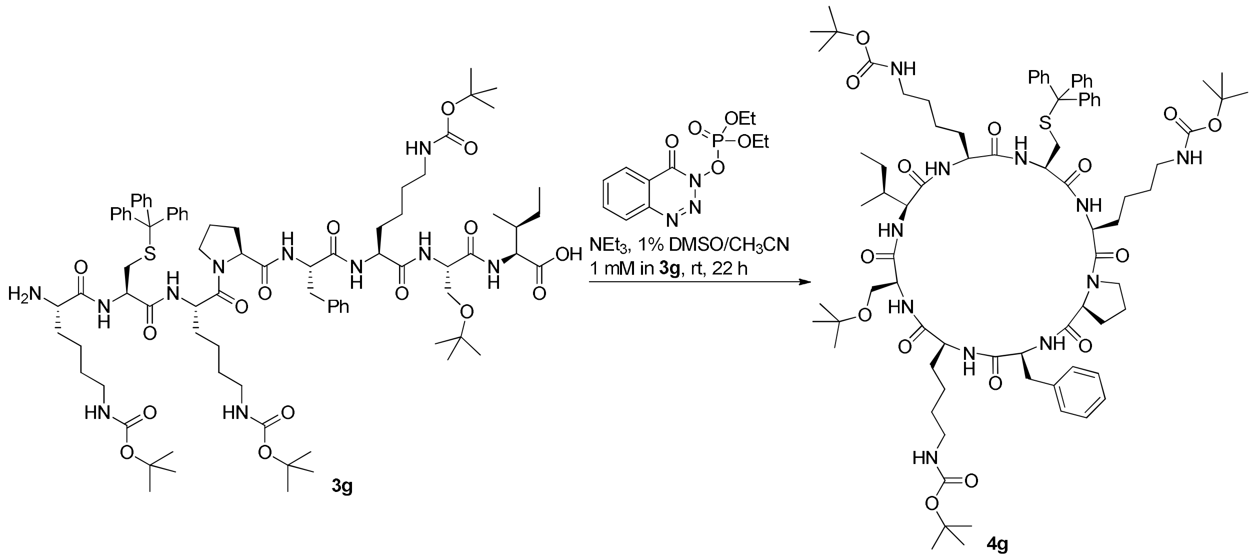

Side chain-protected peptide **3g** (170 mg, 110 µmol, 1 equiv) was subjected to the peptide macrocyclization procedure using scaled amounts of NEt_3 (153 µL, 1.10 mmol), CH_3CN (100 mL), DMSO (1 mL), and DEPBT (65.7 mg, 220 µmol, 2 equiv). Preparatory HPLC method “B for purification ultimately afforded 36.5 mg (MW 1530.95, 23.8 µmol, 22% yield) of protected cyclic peptide **4g**. HPLC-MS characterization using method “G”; retention time: 2.88 min, purity: 94 %, MS (ESI$^+$) *m/z*: $[M+H]^+$ Calcd for $C_{82}H_{120}N_{11}O_{15}S$ 1530.9, found 1530.8.

Product 1g: cyclo-[Lys-Cys-Lys-Pro-Phe-Lys-Ser-Ile]

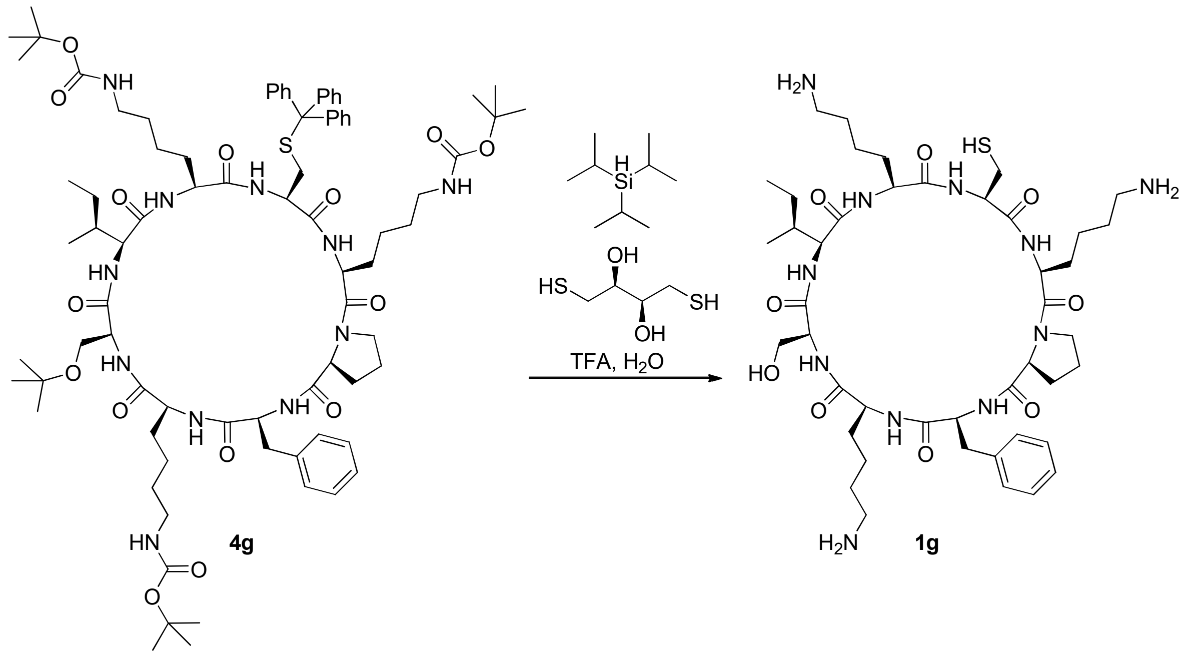

The TFA-mediated deprotection procedure was conducted on peptide **4g** (18 mg, 12 µmol) using scaled amounts of DTT (~1 mg, ~6.5 µmol), H_2O (50 µl), TFA (0.88 mL), and triisopropylsilane (20 µl, 98 µmol). Purification was achieved by preparative HPLC-MS using method "D" to ultimately afford 5.5 mg (tris HCl salt FW: 1041.6, 5.3 µmol, 44% yield) of *cyclo*-[lysinyl-cysteinyl-lysinyl-prolinyl-phenylalaninyl-lysinyl-serinyl-isoleucine] (**1g**) as a solid. UHPLC-HRMS characterization using method "J"; retention time: 15.4 min, HRMS (ESI$^+$) *m/z*: $[M + 2H]^{2+}$ Calcd for $C_{44}H_{75}N_{11}O_9S$ 466.7730, found 466.7737. HCD MS/MS fragment count: Calcd for b/y ions 104, found 47.

Mass chromatogram of **1g** (TIC)

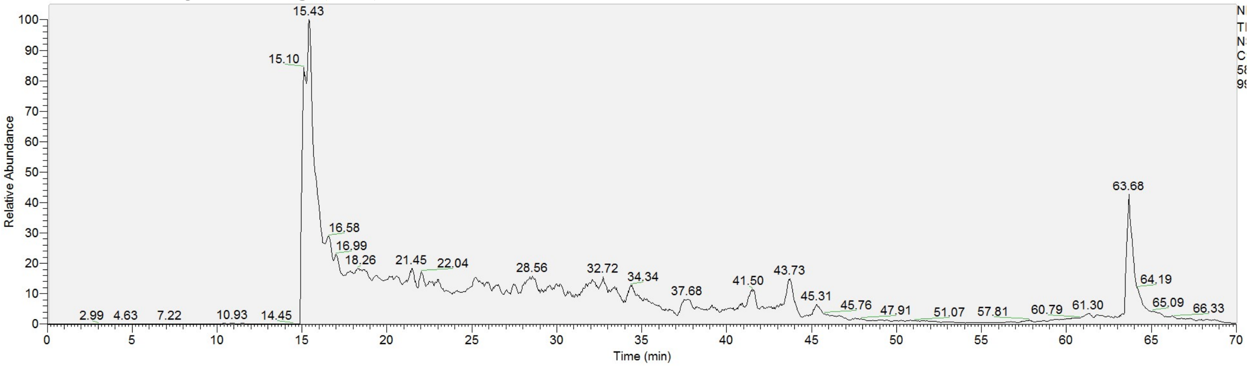

MS expansion illustrating the isotopic profile of **1g** in the doubly-charged ion region (singly-charged ion absent)

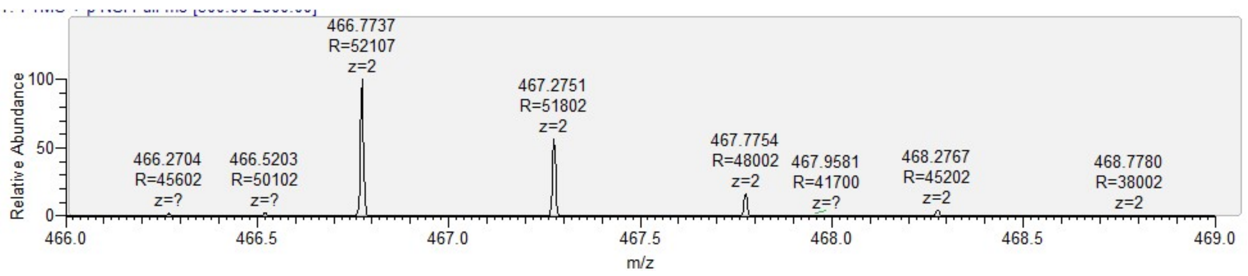

Synthesis of cyclic ELCPPNLL

Intermediate 3h: H-Glu(t-Bu)-Leu-Cys(Trt)-Pro-Pro-Asn(Trt)-Leu-Leu-OH

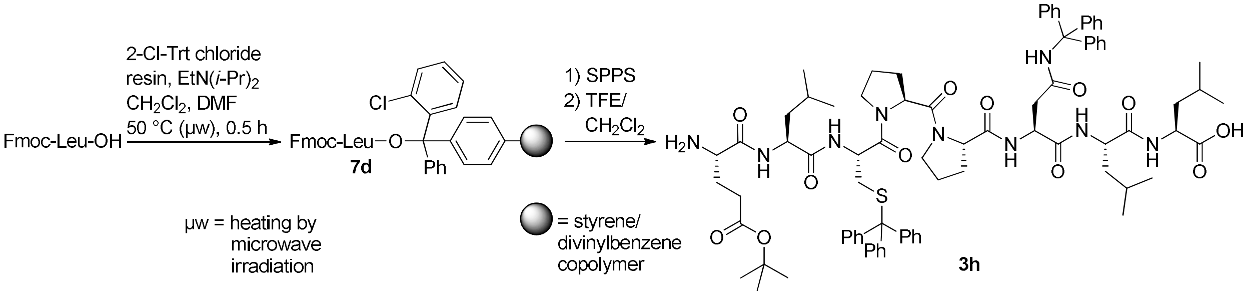

The automated Trt resin loading procedure was executed using a CH_2Cl_2 solution of Fmoc-Leu-OH (5 mL, 0.2 M, 1 mmol) to afford Fmoc-leucinyl Trt resin **7d**. Continuation of SPPS by the automated peptide elongation procedure using standard DMF solutions (5 mL, 0.2 M, 4 equiv.) of Fmoc-Leu-OH, Fmoc-Asn(Trt)-OH, Fmoc-Pro-OH, Fmoc-Pro-OH, Fmoc-Cys(Trt)-OH, Fmoc-Leu-OH, and Fmoc-Glu(*t*-Bu) as input afforded *N*-terminal free amine peptidyl resin **2h**. The latter was subjected to the TFE-mediated Trt resin cleavage procedure using a scaled amount of TFE/ CH_2Cl_2 solvent (5 mL) to afford 168 mg (MW 1438.81, 117 µmol, 47% yield) of side chain-protected peptide **3h**. HPLC-MS characterization using method "G"; retention time: 2.45 min, crude purity: >98 %, MS (ESI$^+$) *m*/*z*: $[M+H]^+$ Calcd for $C_{82}H_{104}N_9O_{12}S$ 1438.8, found 1439.2.

Intermediate 4h: cyclo-[Glu(O-tBu)-Leu-Cys(Trt)-Pro-Pro-Asn(Trt)-Leu-Leu]

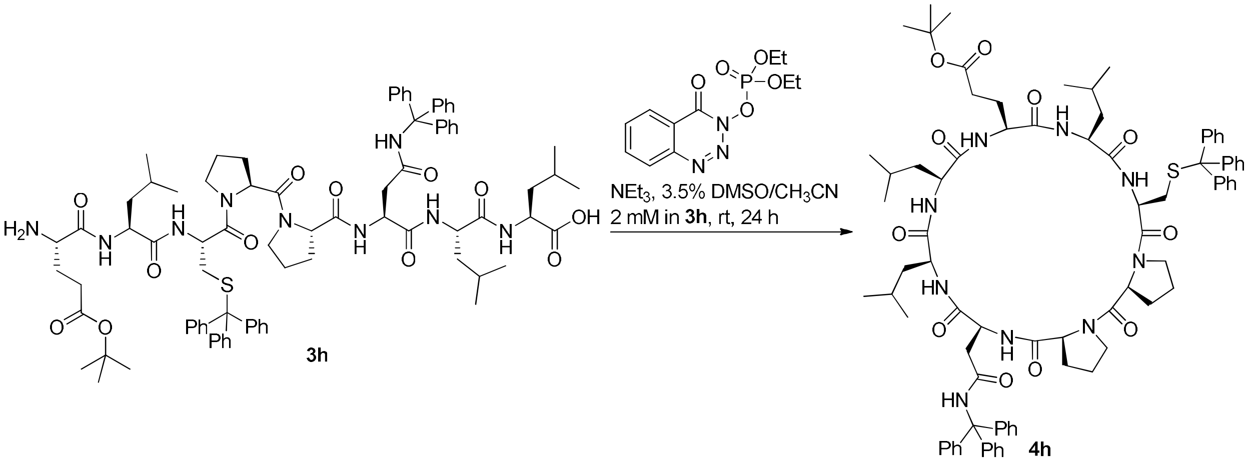

Side chain-protected peptide **3h** (168 mg, 117 μmol) was subjected to the peptide macrocyclization procedure using scaled amounts of NEt_3 (153 μL, 1.17 mmol), CH_3CN (55 mL), DMSO (2 mL), and DEPBT (69.9 mg, 234 μmol, 2 equiv). Preparatory HPLC method "B" for purification, collecting the peak that eluted at 17.99 min, ultimately afforded 44 mg (MW 1420.80, 31 μmol, 26% yield) of protected cyclic peptide **4h**. HPLC-MS characterization using method "G"; retention time: 3.17 min, purity: >98 %.

Product 1h: cyclo-[Glu-Leu-Cys-Pro-Pro-Asn-Leu-Leu]

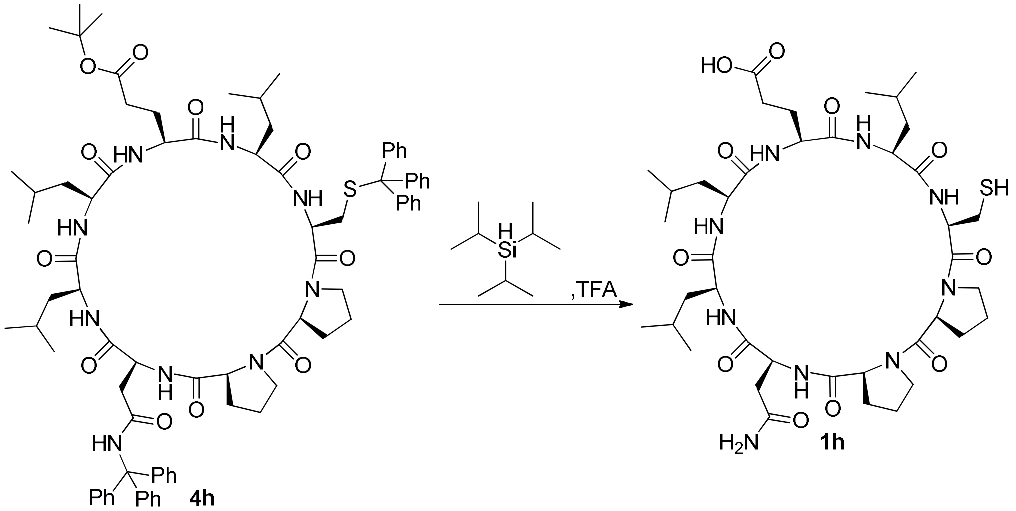

The TFA-mediated deprotection procedure was conducted on peptide **4h** (5 mg, 3.5 μmol), omitting the DTT and H_2O, using adjusted amounts of TFA (1 mL), triisopropylsilane (0.3 mL, 1.5 mmol), and 45 min as reaction time. Purification was achieved by manual reversed-phase chromatography using 50% MeOH in H_2O as eluent to ultimately afford 1 mg (FW: 880.1, 1 μmol, 32% yield) of *cyclo*-[glutamyl-leucinyl-

cysteinyl-prolinyl-prolinyl-aspariginyl-leucinyl-leucine] (**1h**) as a solid. UHPLC-HRMS characterization using method "J"; retention time: 63.9 min, HRMS (ESI$^+$) *m/z*: $[M+H]^+$ Calcd for $C_{40}H_{66}N_9O_{11}S$ 880.4597, found 880.4603. HCD MS/MS fragment count: Calcd for b/y ions 104, found 82.

Mass chromatogram of **1h** (XIC)

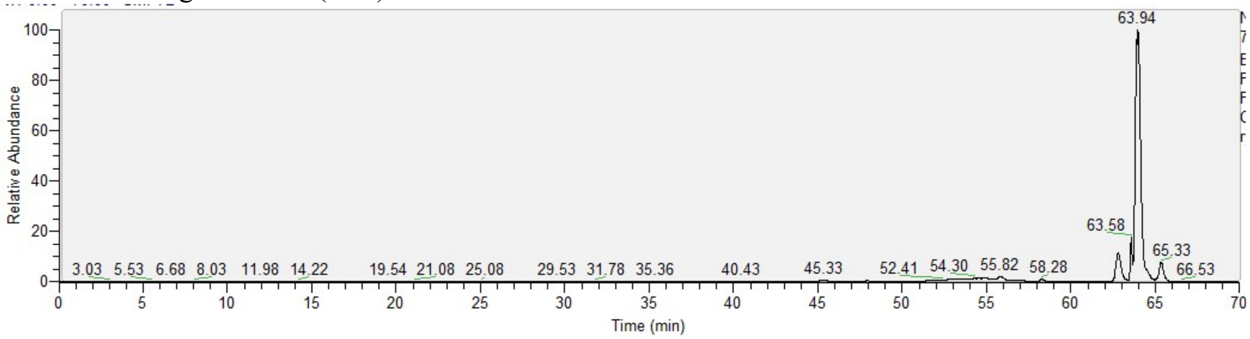

MS expansion illustrating the isotopic profile of **1h**

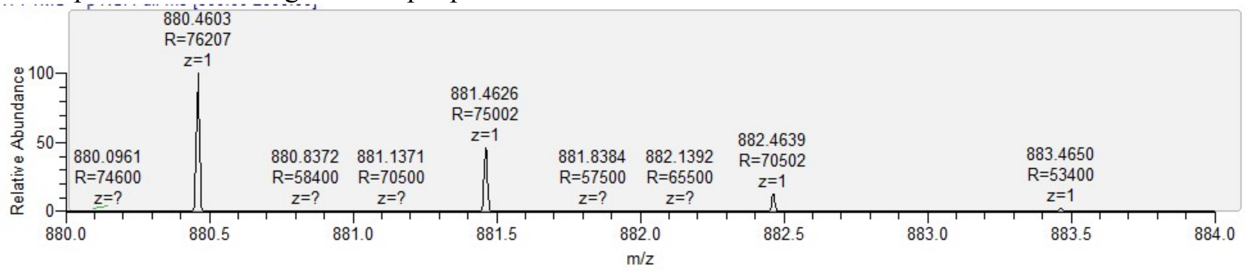

Synthesis of cyclic TKPCPWI

Intermediate 3i: H-Thr(t-Bu)-Lys(Boc)-Pro-Cys(Trt)-Pro-Trp(Boc)-Ile-OH

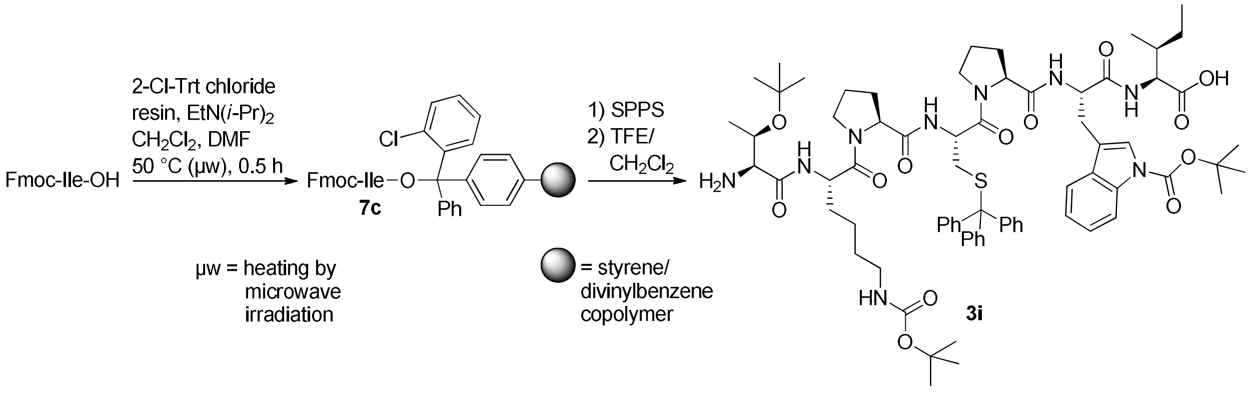

The automated Trt resin loading procedure was executed using a CH_2Cl_2 solution of Fmoc-Ile-OH (5 mL, 0.2 M, 1 mmol) to afford Fmoc-isoleucinyl Trt resin **7c**. Continuation of SPPS by the automated peptide elongation procedure using standard DMF solutions (5 mL, 0.2 M, 4 equiv.) of Fmoc-Trp(Boc)-OH, Fmoc-

Pro-OH, Fmoc-Cys(Trt)-OH, Fmoc-Pro-OH, Fmoc-Lys(Boc)-OH, and Fmoc-Thr(*t*-Bu)-OH as input afforded *N*-terminal free amine peptidyl resin **2i**. The latter was subjected to the TFE-mediated Trt resin cleavage procedure using an adjusted volume of TFE/ CH_2Cl_2 solvent (15 mL) to afford 148 mg (MW 1342.68, 110 μmol, 44% yield) of side chain-protected peptide **3i**. HPLC-MS characterization using method "G"; retention time: 2.40 min, crude purity: 78 %, MS (ESI^+) *m*/*z*: $[M+H]^+$ Calcd for $C_{73}H_{100}N_9O_{13}S$ 1342.7, found 1342.6.

Intermediate 4i: cyclo-[Thr(t-Bu)-Lys(Boc)-Pro-Cys(Trt)-Pro-Trp(Boc)-Ile]

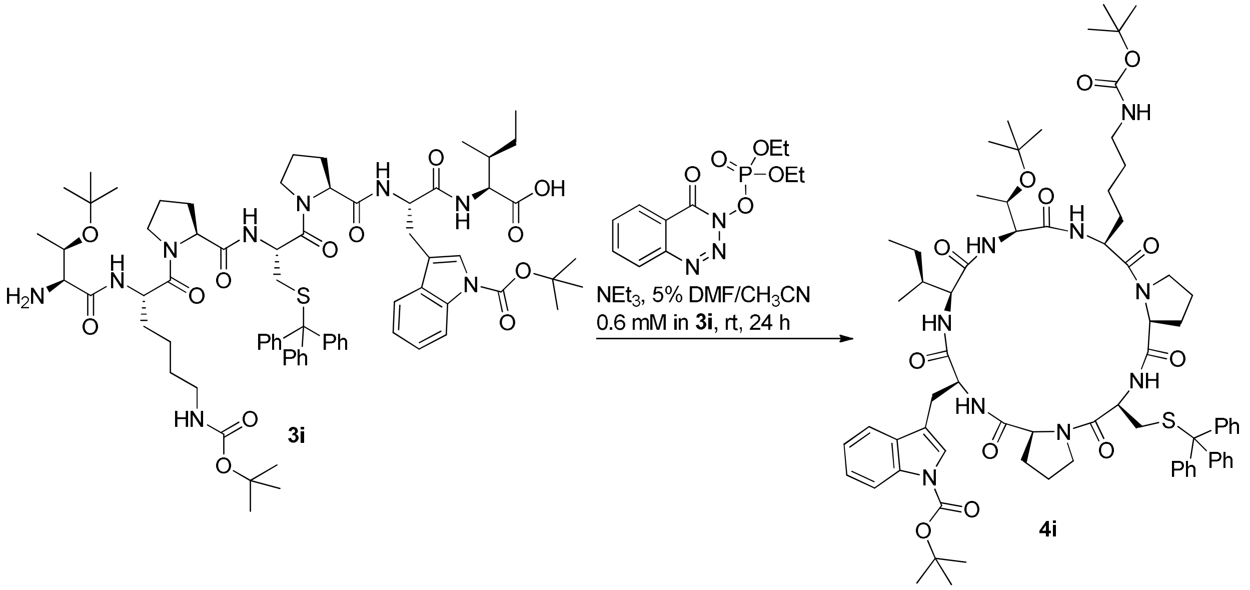

A heterogeneous mixture of side chain-protected peptide **3i** (16.4 mg, 12 μmol), CH_3CN (19 mL), NEt_3 (17 μL, 122 μmol), and DMF (1 mL) was treated with DEPBT (7.3 mg, 24 μmol, 2 equiv) and stirred at room temperature. After 24 h, the reaction was quenched with AcOH, the volume was reduced *in vacuo* to 1-2 mL, and the resulting DMF/CH_3CN solution of the crude was purified by preparatory HPLC using method "C". The fraction that eluted at 8.65 min was concentrated *in vacuo* (2-5 Torr) at 50 °C, then concentrated to dryness with assistance from multiple azeotropic coevaporations with *i*-PrOH to afford 4.6 mg (MW 1324.67, 3.5 μmol, 29% yield) of protected cyclic peptide **4i** as a solid.

Product 1i: cyclo-[Thr-Lys-Pro-Cys-Pro-Trp-Ile]

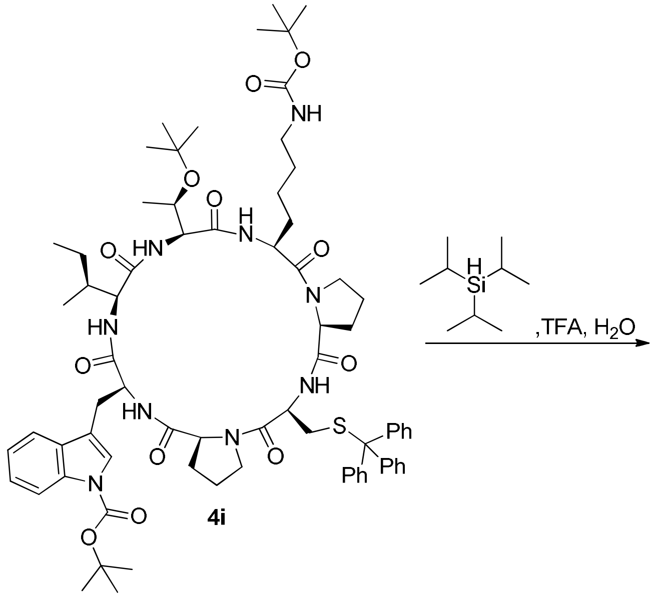

The TFA-mediated deprotection procedure was conducted on peptide **4i** (29.5 mg, 22 µmol), omitting the DTT, using adjusted amounts of H_2O (90 µL), TFA (1.66 mL), triisopropylsilane (90 µL, 445 µmol), and 30 min as reaction time. Purification was achieved by preparative HPLC-MS using method "D" to ultimately afford 7.9 mg (HCl salt FW: 862.48, 9.2 µmol, 41% yield) of *cyclo*-[threoninyl-leucinyl-prolinyl-cysteinyl-prolinyl-tryptophanyl-isoleucine] (**1i**) as a solid. UHPLC-HRMS characterization using method "I"; retention time: 14.5 min, HRMS (ESI$^+$) *m/z*: $[M+H]^+$ Calcd for $C_{4o}H_{60}N_9O_8S$ 826.4280, found 826.4257. HCD MS/MS fragment count: Calcd for b/y ions 77, found 56. Peak tailing in the UHPLC trace was attributed to the presence of slowly equilibrating conformational isomers.

Mass chromatogram of **1i** (TIC)

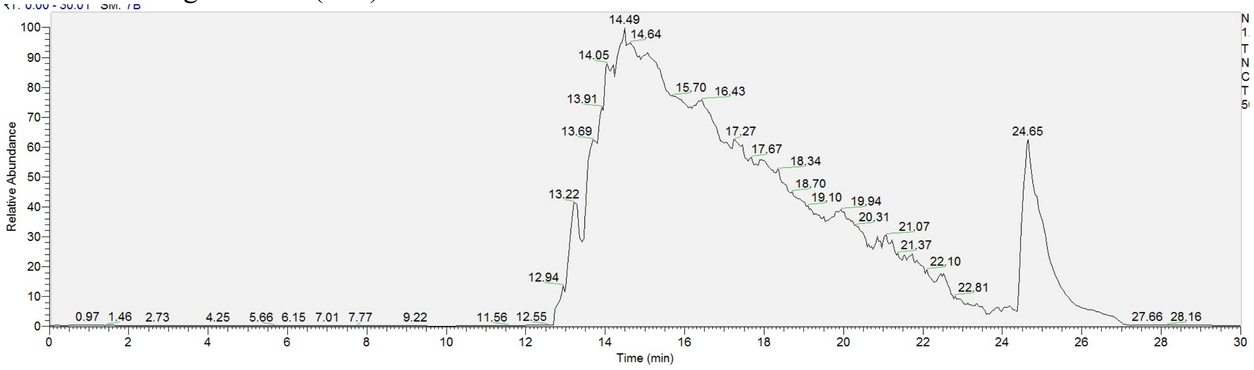

MS expansion illustrating the isotopic profile of **1i**

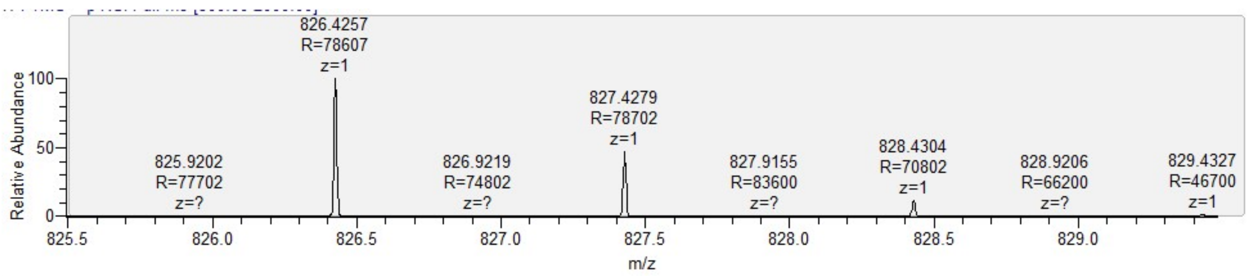

Synthesis of cyclic KSKPCIFK

Intermediate 3j: H-Lys(Boc)-Ser(t-Bu)-Lys(Boc)-Pro-Cys(Trt)-Ile-Phe-Lys(Boc)-OH

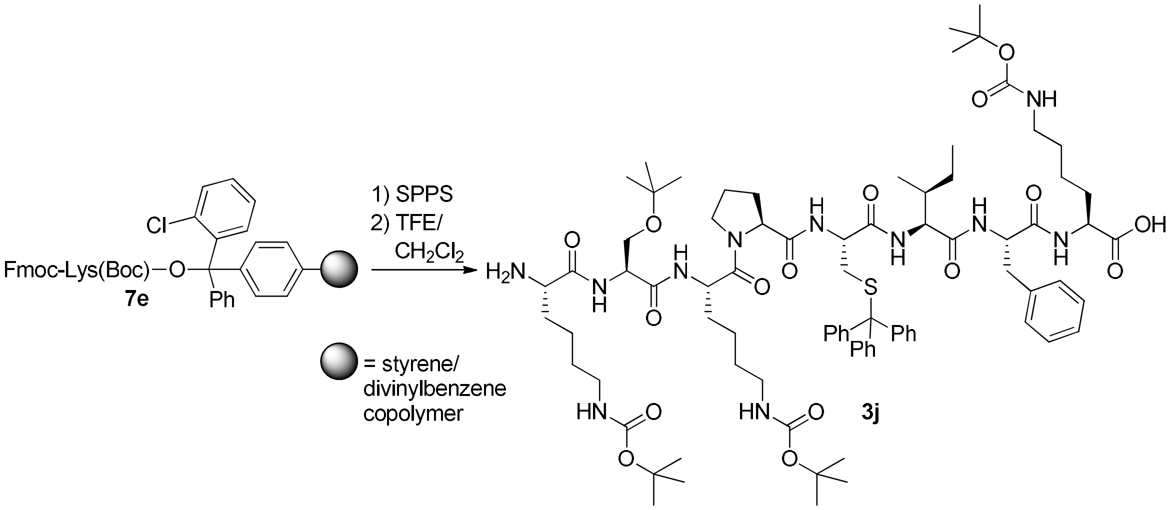

Commercial H-Lys(Boc)-2-Cl-Trt Resin **7e** (465 mg, 0.54 mmol/g 0.250 mmol) was employed in SPPS using the automated peptide elongation procedure with standard DMF solutions (5 mL, 0.2 M, 4 equiv.) of Fmoc-Phe-OH, Fmoc-Ile-OH, Fmoc-Cys(Trt)-OH, Fmoc-Pro-OH, Fmoc-Lys(Boc)-OH, Fmoc-Ser(*t*-Bu)-OH, and Fmoc-Lys(Boc)-OH as input to afford *N*-terminal free amine peptidyl resin **2j**. The latter was subjected to the TFE-mediated Trt resin cleavage procedure using an adjusted volume of TFE/ CH_2Cl_2 solvent (20 mL) to afford 231mg (MW 1548.97, 149 mmol, 60% yield) of side chain-protected peptide **3j**. HPLC characterization using method "H"; retention time: 2.476 min, crude purity: 72%. HPLC-MS characterization using method "G"; retention time: 2.29 min, crude purity: 86%, MS (ESI$^+$) *m*/*z*: $[M+H]^+$ Calcd for $C_{82}H_{122}N_{11}O_{16}S$ 1548.9, found 1548.7.

Intermediate 4j: cyclo-[Lys(Boc)-Ser(t-Bu)-Lys(Boc)-Pro-Cys(Trt)-Ile-Phe-Lys(Boc)]

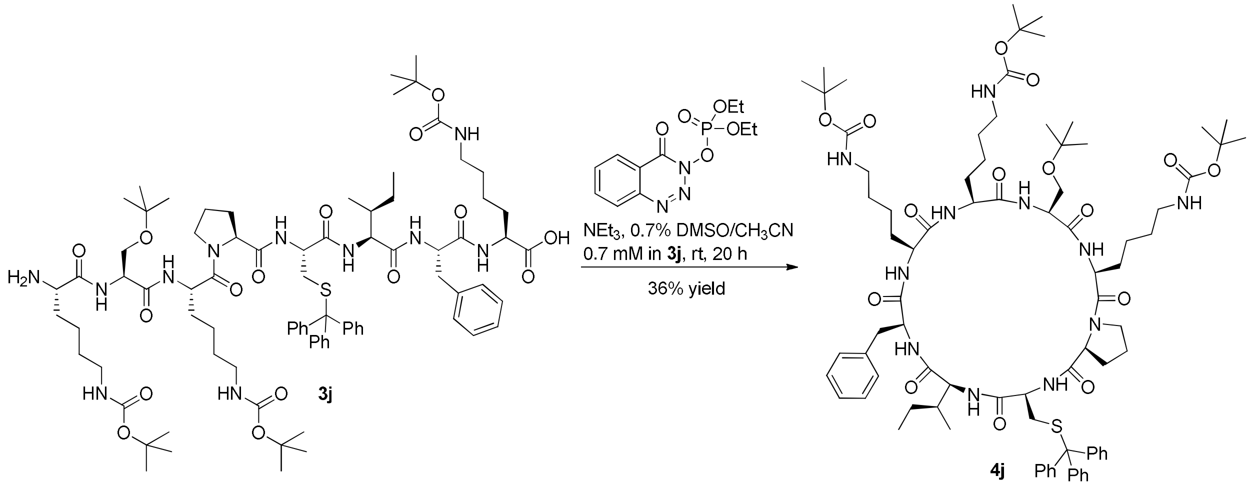

Side chain-protected peptide **3j** (180 mg, 116 µmol, 1 equiv) was subjected to the peptide macrocyclization procedure using adjusted amounts of NEt_3 (162 µL, 1.16 mmol, 1 equiv), CH_3CN (170 mL), DMSO (1.2 mL), and DEPBT (69.5 mg, 232 µmol, 2 equiv). Reaction quenching was achieved using a scaled amount of AcOH (17 mL) while preparatory HPLC purification was conducted according to method "E". The fraction eluting at 8.90 min was collected to ultimately afford 64.5 mg (MW 1530.95, 42.1 µmol, 36% yield) of protected cyclic peptide **4j**. MS (ESI$^+$) *m/z*: $[M+H–Boc]^+$ Calcd for $C_{77}H_{112}N_{11}O_{13}S$ 1430.8, found 1430.8.

Product 1j: cyclo-[Lys-Ser-Lys-Pro-Cys-Ile-Phe-Lys]

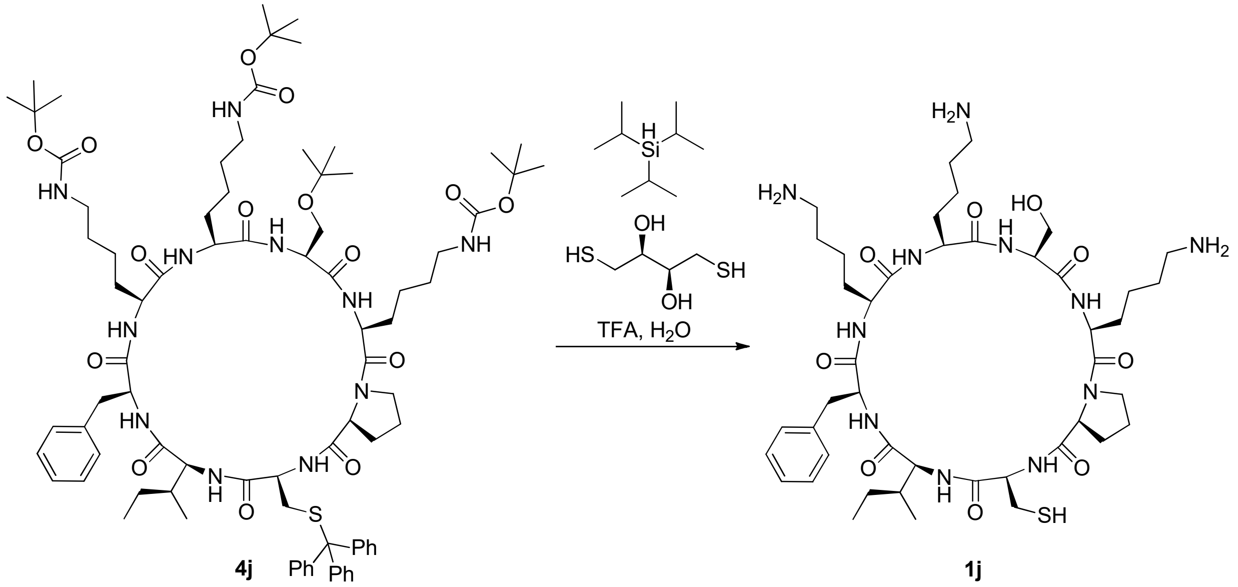

The TFA-mediated deprotection procedure was conducted on peptide **4j** (57 mg, 37 µmol) using scaled amounts of DTT (5.7 mg, 37 µmol), H_2O (0.29 mL), TFA (5.0 mL), and triisopropylsilane (114 µl, 558 µmol). Purification was achieved by preparative HPLC-MS using method "D" to ultimately afford 12.5 mg (tris HCl salt FW: 1041.57, 12.0 µmol, 32% yield) of *cyclo*-[lysinyl-serinyl-lysinyl-prolinyl-cysteinyl-isoleucinyl-phenylalaninyl-lysine] (**1j**) as a solid. UHPLC-HRMS characterization using method "J"; retention time: 15.6 min, HRMS (ESI$^+$) *m/z*: $[M + 2H]^{2+}$ Calcd for $C_{44}H_{75}N_{11}O_9S$ 466.7730, found 466.7730. HCD MS/MS fragment count: Calcd for b/y ions 104, found 61. Disulfide bond dimer **8j** was also identified in the sample; retention time: 15.1 min.

Mass chromatogram of **1j** (XIC)

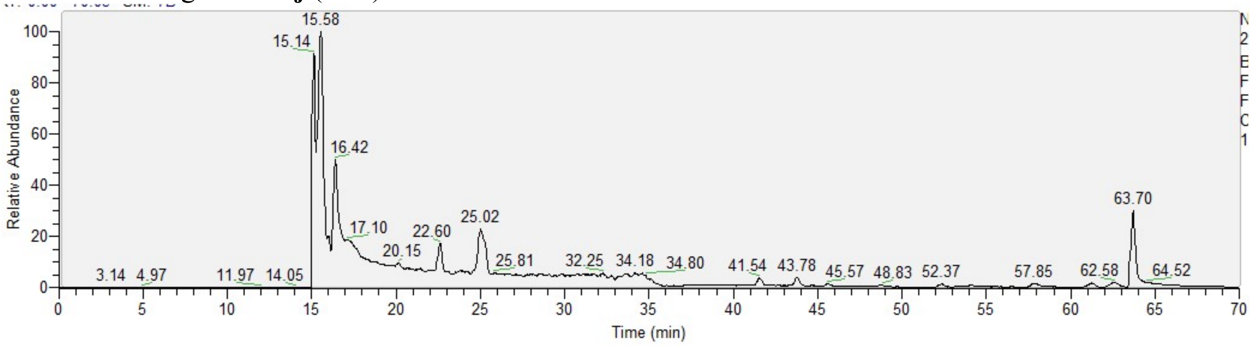

40-99 ACN – 70 min

MS expansion illustrating the isotopic profile of **1j** in the doubly-charged ion region (singly-charged ion absent)

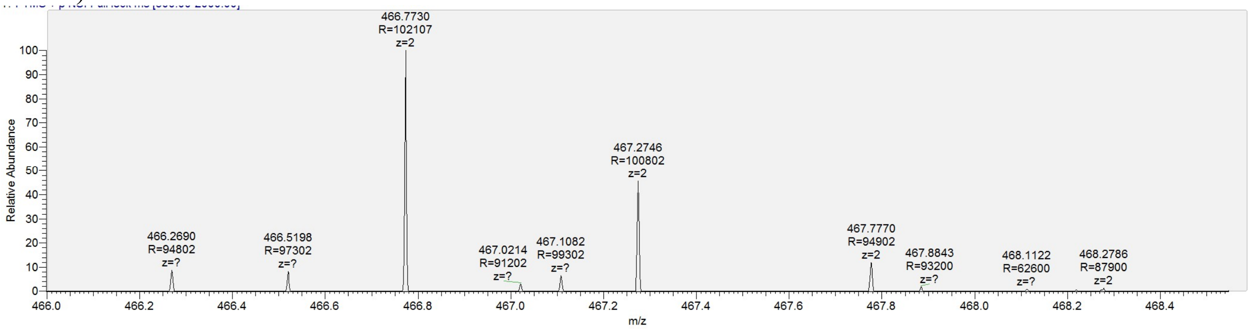

Synthesis of cyclic LEPLNPLC

Intermediate 3k: H-Leu-Glu(t-Bu)-Pro-Leu-Asn(Trt)-Pro-Leu-Cys(Trt)-OH

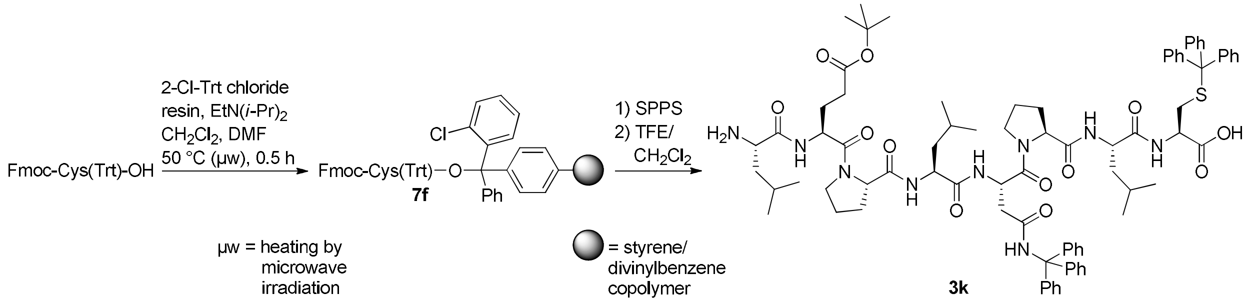

The automated Trt resin loading procedure was executed using a CH_2Cl_2 solution of Fmoc-Cys(Trt)-OH (5 mL, 0.2 M, 1 mmol) to afford Fmoc-Cysteinyl Trt resin **7f**. Continuation of SPPS by the automated peptide elongation procedure using standard DMF solutions (5 mL, 0.2 M, 4 equiv.) of Fmoc-Leu-OH, Fmoc-Pro-OH, Fmoc-Asn(Trt)-OH, Fmoc-Leu-OH, Fmoc-Pro-OH, Fmoc-Glu(*t*-Bu)-OH, and Fmoc-Leu-OH as input afforded *N*-terminal free amine peptidyl resin **2k**. The latter was subjected to the TFE-mediated Trt resin cleavage procedure to afford 155 mg (FW 1438.81, 108 μmol, 43% yield) of side chain-protected peptide **3k**. HPLC-MS characterization using method "G"; retention time: 2.52 min, crude purity: 62 %, MS (ESI$^+$) *m*/*z*: $[M+H]^+$ Calcd for $C_{82}H_{104}N_9O_{12}S$ 1438.8, found 1438.6.

Intermediate 4k: cyclo-[Leu-Glu(t-Bu)-Pro-Asn(Trt)-Pro-Leu-Cys(Trt)]

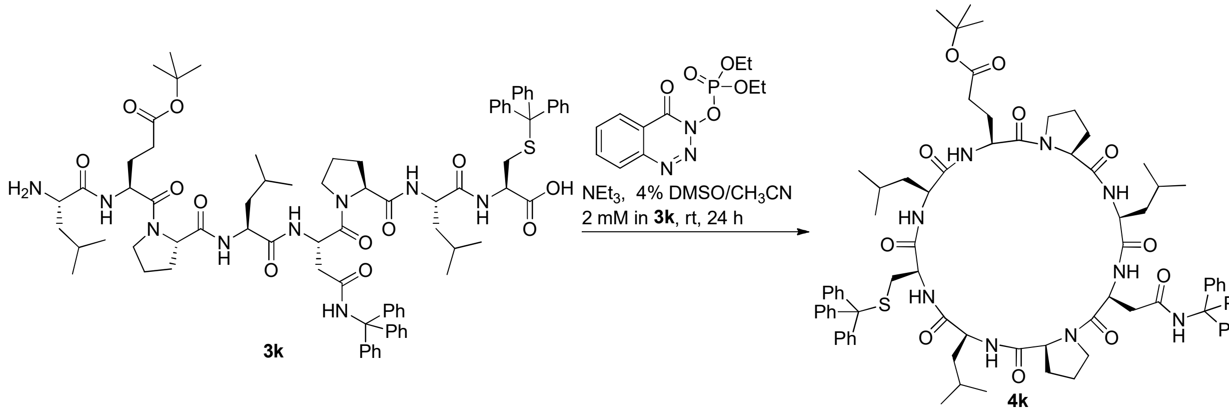

Side chain-protected peptide **3k** (155 mg, 108 μmol) was subjected to the peptide macrocyclization procedure using scaled amounts of NEt_3 (141 μL, 1.08 mmol), CH_3CN (50 mL), DMSO (2 mL), and DEPBT (64.5 mg, 215 μmol, 2 equiv). Preparatory HPLC method "C" for purification ultimately afforded 36 mg (MW 1420.80, 25 μmol, 23% yield) of protected cyclic peptide **4k**. HPLC-MS characterization using method "G"; retention time: 3.36 min, purity: >99 %. HPLC-MS, MS (ESI$^+$) *m*/*z*: $[M+H]^+$ Calcd for $C_{82}H_{102}N_9O_{11}S$ 1420.7, found 1420.6.

Product 1k: cyclo-[Leu-Glu-Pro-Leu-Asn-Pro-Leu-Cys]

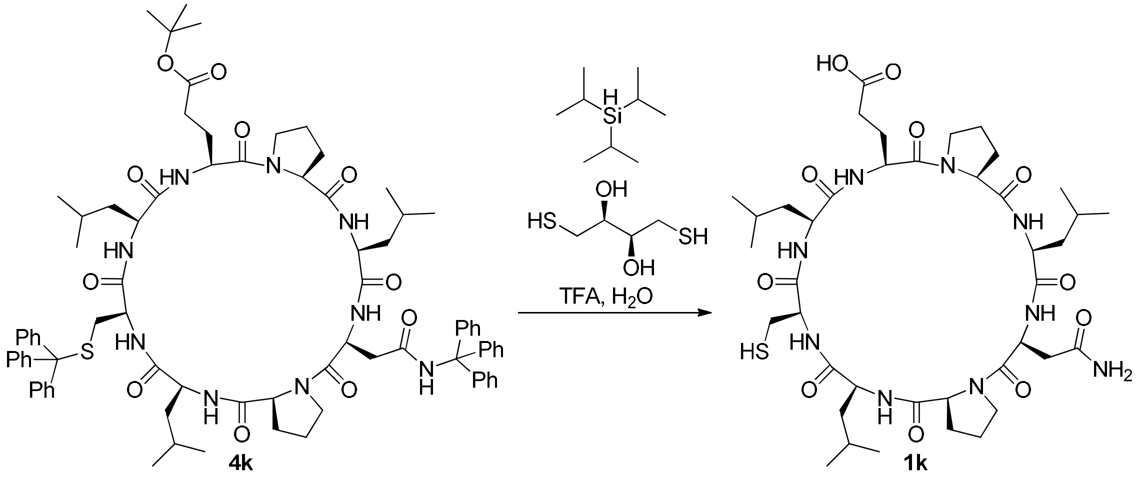

The TFA-mediated deprotection procedure was conducted on peptide **4k** (36 mg, 25 µmol) using scaled amounts of DTT (~1 mg, ~6.5 µmol), H_2O (0.1 mL), TFA (1.9 mL), and triisopropylsilane (104 µl, 507 µmol). Purification was achieved by preparative HPLC-MS using method "D" to ultimately afford 10.9 mg (MW 880.06, 12.4 µmol, 49% yield) of *cyclo*-[leucinyl-glutamyl-prolinyl-leucinyl-asparaginyl-prolinyl-leucinyl-cysteine] (**1k**) as a solid. UHPLC-HRMS characterization using method "I"; retention time: 14.0, 16.7 min, HRMS (ESI$^+$) *m/z*: $[M+H]^+$ Calcd for $C_{40}H_{66}N_9O_{11}S$ 880.4597, found 880.4572. HCD MS/MS fragment count: Calcd for b/y ions 104, found 74. The presence of two broad peaks in UHPLC trace was attributed to the presence of slowly equilibrating conformational isomers.

Mass chromatogram of **1k** (XIC)

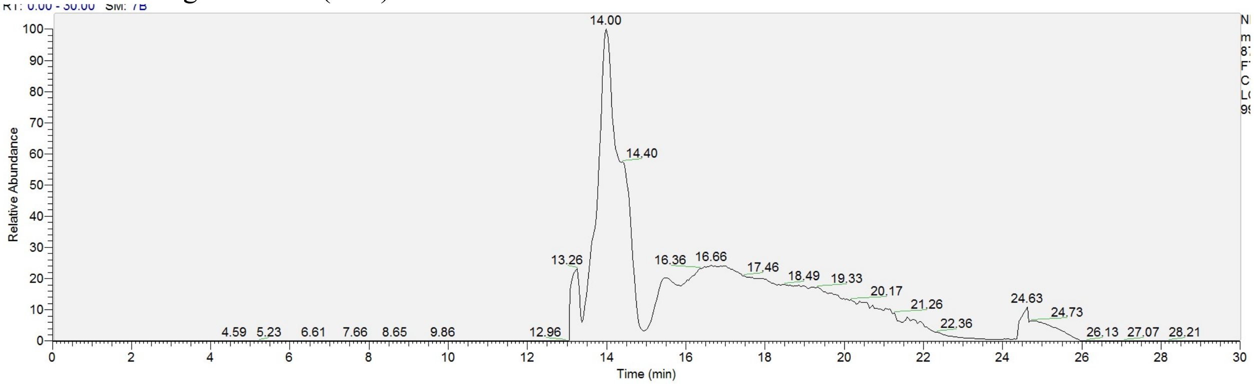

MS expansion illustrating the isotopic profile of **1k**

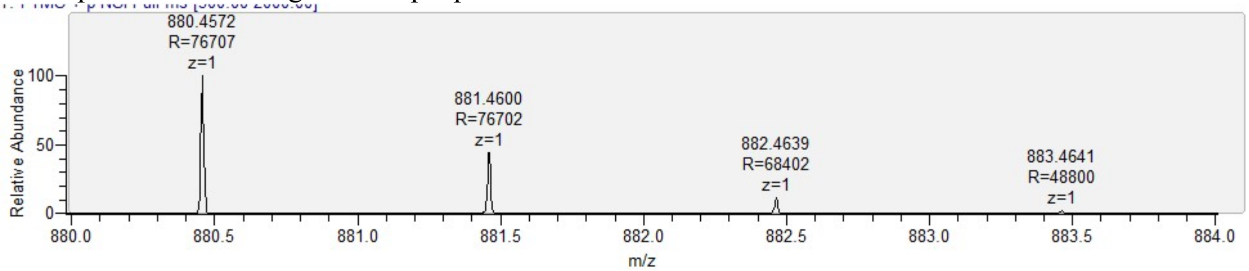

III. References

1. V. Krchnak and J. Vagner, *Peptide Research*, 1990, **3**, 182-193.
2. E. Kaiser, Colescot.Rl, Bossinge.Cd and P. I. Cook, *Anal. Biochem.*, 1970, **34**, 595-&.
3. J. M. Collins and M. J. Collins, *Biopolymers*, 2003, **71**, 361-361.
4. C. Echalier, S. Al-Halifa, A. Kreiter, C. Enjalbal, P. Sanchez, L. Ronga, K. Puget, P. Verdie, M. Amblard, J. Martinez and G. Subra, *Amino Acids*, 2013, **45**, 1395-1403.
5. L. Wang, N. Gagey-Eilstein, S. Broussy, M. Reille-Seroussi, F. Huguenot, M. Vidal and W. Q. Liu, *Molecules*, 2014, **19**, 15391-15407.
6. S. L. Pedersen and K. J. Jensen, *Chapter 3: Peptide release, side-chain deprotection, work-up, and isolation, in Peptide synthesis and applications*, 2013, **1047**, 43-63.
7. P. Roepstorff and J. Fohlman, *Biomedical Mass Spectrometry*, 1984, **11**, 601-601.
